# The potential of acupuncture in treating sarcopenia: a systematic review and meta-analysis of randomized controlled trials

**DOI:** 10.3389/fpubh.2025.1696030

**Published:** 2025-11-10

**Authors:** Xingchen Niu, Dingding Zhang, Tian Gao, Yong Zhang, Dan Zhang, Donghai Li, Ping Zeng

**Affiliations:** 1 Innovation Research Institute of Traditional Chinese Medicine, Shandong University of Traditional Chinese Medicine, Jinan, China; 2 State Key Laboratory for Complex, Severe, and Rare Diseases, Institute of Basic Medical Sciences, Chinese Academy of Medical Sciences and School of Basic Medicine, Peking Union Medical College, Beijing, China; 3 Center for Prevention and Early Intervention, National Infrastructures for Translational Medicine, Institute of Clinical Medicine, Peking Union Medical College Hospital, Chinese Academy of Medical Science and Peking Union Medical College, Beijing, China; 4 Experimental Center, Shandong University of Traditional Chinese Medicine, Key Laboratory of Traditional Chinese Medicine Classical Theory, Ministry of Education, Shandong University of Traditional Chinese Medicine, Jinan, Shandong Province, China; 5 Department of Burn, Plastic & Reconstructive Surgery, and Wound Repair, Beijing Fengtai You'anmen Hospital, Beijing, China; 6 Health and Medical Department, Peking Union Medical College Hospital, Chinese Academy of Medical Science, Beijing, China

**Keywords:** sarcopenia, acupuncture, systematic review, meta-analysis, non-pharmacological therapy

## Abstract

The global population is aging at an unprecedented rate, leading to a sharp rise in late-life diseases such as sarcopenia. Despite its increasing prevalence, effective pharmacological treatments, exercise regimens, and dietary recommendations for sarcopenia remain limited. Acupuncture has demonstrated potential in improving quality of life for individuals with sarcopenia. However, whether acupuncture is effective for treating sarcopenia remains unclear. This systematic review and meta-analysis aim to assess the therapeutic effectiveness of acupuncture in treating sarcopenia. We conducted a comprehensive search across nine major English and Chinese electronic databases, covering all available studies up to May 2024. We compared sarcopenia outcomes between patients who received acupuncture and those who did not. The retrieved literature was systematically organized and categorized using EndNote 21. We performed data analysis using Review Manager 5.3 and STATA 18.0. Our comprehensive search identified 1,106 publications, including 384 basic research, 324 systematic reviews and meta-analyses, 281 unrelated studies, 109 randomized controlled trials, and 8 case reports. Finally we analyzed 10 RCTs. Compared to conventional treatment, acupuncture significantly improved total treatment efficiency (RR = 1.40, 95% CI: 1.13–1.73), muscle mass (SMD = 1.31, 95% CI: 0.19–2.43), grip strength (SMD = 0.66, 95% CI: 0.49–0.83), and usual gait speed (SMD = 2.53, 95% CI: 0.78–4.28). Additionally, acupuncture reducing C-reactive protein levels (SMD = −0.99, 95% CI: −1.42–−0.56). Among the results, acupuncture-based therapies demonstrated therapeutic potential on muscle mass and some physical function in patients with sarcopenia. Moreover, acupuncture constitutes an essential complementary modality for frail older adults unable to tolerate conventional treatments. However, the limited number and quality of RCTs necessitates further research to confirm these findings and fully elucidate acupuncture’s potential in sarcopenia management. Further validation through multicenter, high-quality RCTs with larger sample sizes is essential to ensure the robustness and generalizability of these findings.

**Systematic review registration:**

https://www.crd.york.ac.uk/PROSPERO/view/CRD420251040930, Identifier, CRD420251040930.

## 1 Introduction

Sarcopenia, first introduced by Professor Irwin Rosenberg in 1989, originates from the Greek words for “muscle” and “loss.” It describes an age-related decline in skeletal muscle mass and function, primarily marked by reductions in muscle strength and mass (1). Between ages 50 and 70, muscle mass decreases by approximately 8% per decade, with the decline accelerating to 10–15% after age 70 (2). The prevalence of sarcopenia varies widely depending on the diagnostic criteria used. In 2010, the European Working Group on Sarcopenia in Older People (EWGSOP) established a consensus definition, classifying sarcopenia as a progressive, generalized syndrome characterized by the loss of skeletal muscle mass, strength, and physical function. This condition contributes to impaired mobility, reduced quality of life, and increased mortality risk (3).

Clinically, sarcopenia manifests as reduced muscle mass, diminished grip strength, and impaired physical function. In Traditional Chinese Medicine (TCM), similar symptoms are observed in Fleshy Wilting and Consumptive Disease, conditions that present as a slender body build, muscle atrophy, and generalized weakness, closely aligning with the clinical features of sarcopenia.

The development of effective pharmacological therapies for sarcopenia has progressed slowly (4). Currently, the most effective strategies involve resistance training combined with optimized nutritional support (5). Older adults, particularly those who are frail, are encouraged to increase protein intake and consider dietary supplements. While exercise benefits muscle health, many older individuals face physical limitations that restrict their ability to engage in regular training. No specific medications have been approved for sarcopenia treatment, although some drugs offer modest improvements in muscle mass and strength (6). However, these medications often cause significant side effects, highlighting the urgent need for safer and more targeted therapeutic options.

Acupuncture offers unique advantages, with its clinical efficacy validated over millennia of practice. It is easy to perform and typically has a low occurrence of adverse effects, which makes it a cost-effective treatment method. It can quickly alleviate clinical symptoms, promote patients’ functional recovery, and help maintain their health. These unique characteristics render acupuncture a valuable approach for preventing, treating, and rehabilitating diseases. Increasing research supports the significant efficacy of acupuncture in treating sarcopenia. For example, acupuncture enhances muscle function by stimulating specific acupoints to regulate Qi and blood circulation.

It is important to note that in real-world clinical practice of treatment, acupuncture is often employed as part of a multimodal therapeutic strategy, particularly for complex geriatric syndromes like sarcopenia. This review therefore aims to evaluate the overall effect of acupuncture as it is commonly practiced, including studies where it is used alone or combined with other conventional therapies. This approach provides a more pragmatic assessment of its potential role in clinical management.

Acupuncture can complement Western medicine, offering a synergistic approach to sarcopenia treatment. By enhancing digestive function, improving nutrient absorption, and modulating immune responses, acupuncture helps optimize the muscle microenvironment while exerting anti-inflammatory and antioxidant effects (7). These mechanisms collectively slow aging, reduce muscle atrophy, and improve physical function (8). Integrating acupuncture with Western medical treatments not only enhances therapeutic outcomes but also minimizes potential side effects. Thus, acupuncture presents a promising strategy for sarcopenia management.

While preliminary evidence from preclinical studies and small-scale clinical trials suggests that acupuncture may alleviate sarcopenia through mechanisms such as anti-inflammatory action, metabolic modulation, and neuromuscular adaptation, the current body of evidence is fragmented and hindered by methodological inconsistencies. Existing research is limited by insufficient sample sizes, variability in intervention protocols (e.g., acupoint selection, stimulation parameters), and a lack of robust translational data bridging animal-derived molecular pathways (e.g., PI3K/Akt/mTOR activation) to human clinical outcomes. Furthermore, the synergistic potential of acupuncture combined with established interventions like resistance training remains underexplored in multi-arm trials. To address these gaps, this systematic review and meta-analysis rigorously synthesize high-quality evidence, employing sensitivity analyses to mitigate heterogeneity and the GRADE framework to evaluate evidence reliability. By clarifying subgroup-specific efficacy and safety profiles, this work aims to establish a standardized evidence base for integrating acupuncture into multimodal sarcopenia management strategies, ultimately guiding clinical decision-making and optimizing therapeutic outcomes.

## 2 Methods

### 2.1 Study registration

We prospectively registered this systematic review and meta-analysis on the International Prospective Register of Systematic Reviews (PROSPERO) under registration number CRD420251040930. The study strictly adhered to the Preferred Reporting Items for Systematic Reviews and Meta-Analyses (PRISMA) Protocols guidelines (9). Additionally, we structured our methodology based on the updated PRISMA 2020 statement for systematic review reporting (10) and the Cochrane Handbook for Systematic Reviews of Interventions (11), ensuring a rigorous and transparent review process (12).

Inclusion and exclusion criteria.

Studies that met all of the following criteria were included:

1. Study Design: Randomized controlled trials (RCTs).
2. Patient Population: Participants diagnosed with sarcopenia according to any established criteria, including the EWGSOP (3), the International Working Group on Sarcopenia (IWGS) (13), the Asian Working Group for Sarcopenia (AWGS) (14), domestic guidelines, or clinical experience (15, 16).
3. Intervention: Acupuncture treatment.
4. Outcomes: Total treatment efficacy, body composition, physical function, or related biomarkers.
5. Language: Studies published in Chinese or English.

Studies that met any of the following criteria were excluded:

1. Study Design: non-RCT studies.
2. Patient Population: Studies that did not diagnose participants with sarcopenia.
3. Intervention: Studies that did not include acupuncture treatment.
4. Study Quality: Studies of research poor methodological quality or those lacking outcome data, even after contacting the original authors.
5. Language: Studies published in languages other than Chinese or English.

Two independent reviewers (Xingchen Niu and Tian Gao) sequentially screened studies based on their titles, abstracts, and full texts to assess eligibility. To ensure comprehensive retrieval of relevant studies, we conducted additional searches beyond electronic databases, including gray literature, clinical trial registries, and reference lists of related publications and review articles. After retrieving the literature, we used EndNote software to identify and remove duplicates. We then screened the titles and abstracts of all remaining records against the predefined inclusion and exclusion criteria to determine their relevance. We are confident that our search strategy was sufficiently sensitive to minimize the risk of omitting significant studies. Given the anticipated volume of eligible studies and the broad scope of our review, it is unlikely that undetected studies would meaningfully alter our overall findings.

### 2.2 Search strategy

Two independent reviewers (Xingchen Niu and Tian Gao) conducted the literature search according to a predefined strategy across nine electronic databases: PubMed, Web of Science, Embase, Cochrane Library, Scopus, Chinese Biomedical Literature Database (SinoMed), Chinese National Knowledge Infrastructure (CNKI), Wan Fang database, and VIP database. The search covered all available records from the inception of each database through May 2024.

### 2.3 Selection of studies

The PICOS strategy was defined as follows:

1. P (Patient): Individuals diagnosed with sarcopenia, regardless of age, gender, or race.
2. I (Intervention): Acupuncture treatment.
3. C (Comparison): Comparison with a control group without acupuncture.
4. O (Outcome): Relevant indicators for evaluating body composition, physical function, and biomarkers.
5. S (Study Design): All study designs including RCTs.

### 2.4 Bias and quality assessment risks of evidence certainty

Following PRISMA guidelines, two independent reviewers (Xingchen Niu and Tian Gao) assessed the methodological quality and risk of bias for the included studies. We used the revised Cochrane Risk of Bias 2 Tool (17) to evaluate bias across the following domains:

1. Risk of bias due to randomization
2. Bias resulting from deviations from intended interventions
3. Bias due to missing outcome data
4. Bias in outcome measurement
5. Bias in the selection of reported results
6. Other potential sources of bias

Each domain was classified into one of three categories: high risk of bias, some concerns, or low risk of bias. The overall quality of evidence was assessed using the Grading of Recommendations, Assessment, Development, and Evaluation (GRADE) guidelines (18). Any discrepancies between the two reviewers were resolved by consensus, or, if necessary, by consulting a third reviewer (Ping Zeng), who acted as an arbitrator.

### 2.5 Data extraction

Studies that met the inclusion criteria underwent a comprehensive review and quality assessment before data extraction. Key details, including author, publication year, participant country, diagnostic criteria, sample size, age range, and intervention types, were systematically gathered.

Two independent reviewers (Xingchen Niu and Tian Gao) extracted data on predefined primary and secondary outcomes using standardized tables developed by the authors to ensure consistency and accuracy (19). When multiple publications reported on the same trial, the most recent or comprehensive report was selected for data extraction. Any disagreements about study eligibility or data interpretation were resolved through discussion or, if necessary, by consulting a third reviewer (Ping Zeng), who acted as an arbitrator. If additional information was required for study inclusion, the original authors were contacted for clarification. Studies with insufficient or inaccessible data were evaluated for their potential impact based on the available information. If the data were deemed inadequate for analysis, the study was excluded from the review.

### 2.6 Statistical analysis

The effects of acupuncture interventions were evaluated based on four key outcomes: total efficiency, changes in body composition, physical function, and related biomarkers. These outcomes were measured from baseline to the end of the intervention period. Total efficiency was analyzed as a dichotomous variable, with results reported as relative risk (RR) and 95% confidence intervals (CIs) to quantify uncertainty and assess the effects of acupuncture interventions. Continuous data, such as changes in body composition, physical function, and related biomarkers, were analyzed using standardized mean differences (SMD). Effect sizes were interpreted according to Cohen’s guidelines for SMDs: 0.2 represents a small effect, 0.5 a moderate effect, and 0.8 a large effect (20). Forest plots were generated to visually present the intervention effect estimates and their corresponding 95% CIs. Statistical analyses were performed using Review Manager (version 5.3) and Stata (version 18.0).

To assess publication bias, funnel plots were created when more than 10 studies were included in the analysis. Asymmetry in these plots was tested using Egger’s test, with a *p*-value < 0.05 indicating significant publication bias. Heterogeneity among studies was evaluated using the χ^2^ test, with a significance level set at *α* = 0.1. The degree of inconsistency was measured using the I^2^ statistic (21). When conducting the data analysis, given heterogeneity in acupoint selection across trials, all pooled analyses were performed under a random-effects model.

## 3 Results

### 3.1 Study selection

**Figure 1:**
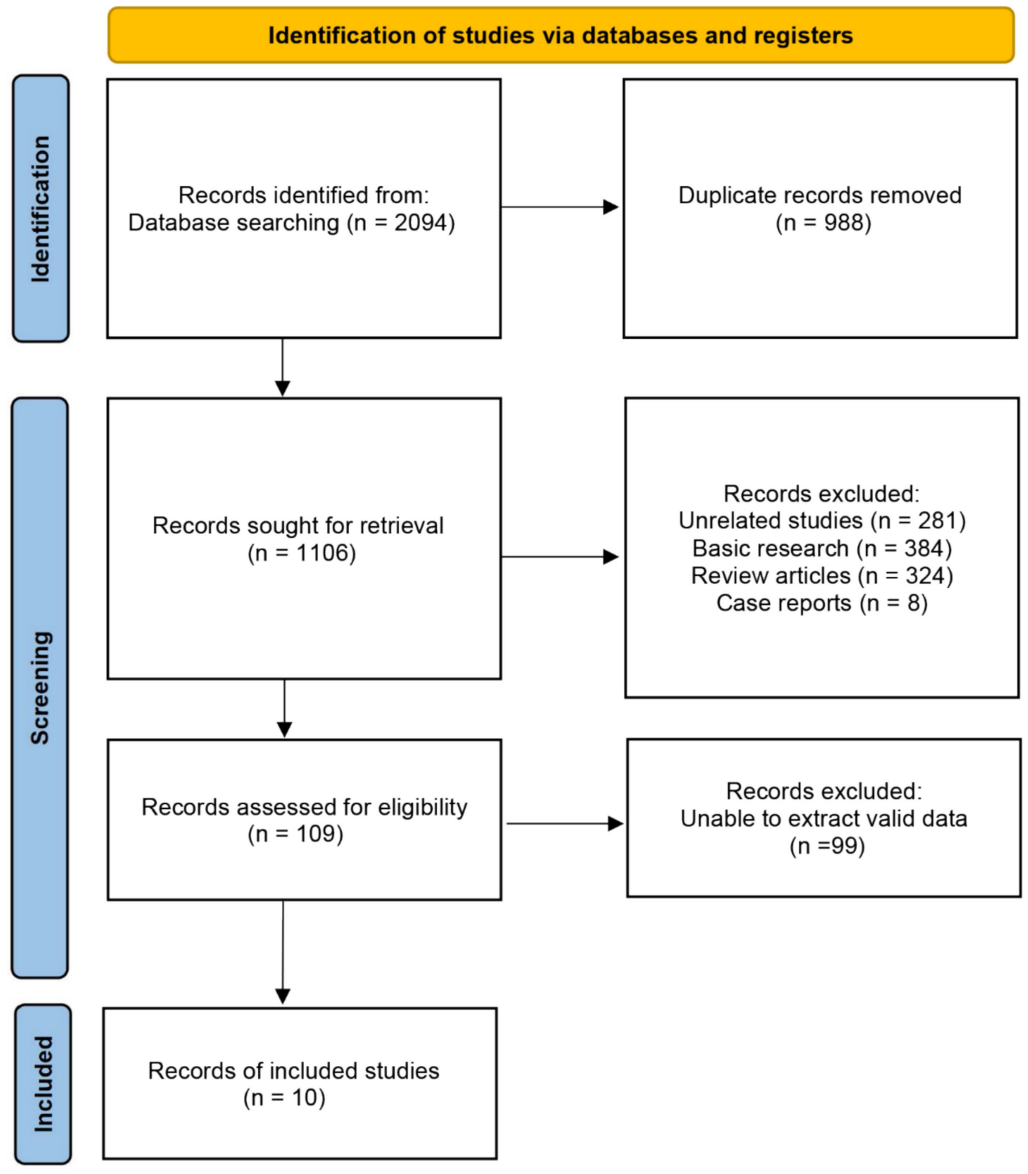
PRISMA flow diagram.

The PRISMA flow diagram in Figure 1 which outlines the search process and results. Our initial electronic search identified 2,094 records. After removing 988 duplicates, 1,106 records remained for title and abstract screening. We excluded studies for the following reasons: unrelated topics (*n* = 281), basic research (*n* = 384), review articles (*n* = 324), and case reports (*n* = 8). This screening left 109 studies for full-text review to assess eligibility. We excluded 99 studies due to poor methodological quality or insufficient data. Ultimately, 10 studies met the inclusion criteria and were included in our analysis.

### 3.2 Study characteristics

Table 1 summarizes the characteristics of the included studies. We analyzed 10 RCTs (22–31) involving 754 participants. These studies, conducted between 2018 and 2024, included sample sizes ranging from 15 to 260 participants, with ages spanning 45 to 85 years. Most studies were conducted in Asia, while one originated from South America. Regarding diagnostic criteria, 6 studies (60%) applied the AWGS criteria, 1 study (10%) used the EWGSOP criteria, 1 studies (10%) followed the IWGS criteria, while 2 studies (20%) used the Chinese Expert Consensus on Diagnosis and Treatment for Elderly. Regarding acupoint selection, ST36 was most frequently employed (*n* = 9), followed by SP6 and LI11 (*n* = 7), GB34 (*n* = 6), ST31, KI3, ST34, LI4, and LI14 (*n* = 5), LI10 (*n* = 3), ST32, SP9, LR3, ST37, ST39, CV4, ST25, ST40, and CV12 (*n* = 2), and LI15, SJ5, SP3, SI3, SJ13, GB31, SP10, BL57, BL60, KI1, ST41, CV6, CV14, ST44, ST35, and LU5 (*n* = 1).

**Table 1:** Main characteristics of included studies.

Author/Year	Country	Diagnostic criteria	Sample size (EG/CG)	Mean Age (EG/CG, years)	Intervention EG	Intervention CG
Feng et al. (22)	China	CECDTES 2021	130/130	56 ± 11/57 ± 11	Acupuncture (LI4, SI3, SJ5, LI10, LI11, LI14, SJ13, GB31, ST34, SP10, ST36, GB34, SP9, ST40, BL57, SP6, BL60, KI3, LR3, KI1) combined with conventional treatment	Conventional treatment
Soares Mendes Damasceno et al. (32)	Brazil	EWGSOP 2010	11/4	72 ± 7.9/63.5 ± 3.3	Acupuncture (LI4, KI3, SP6, GB34, ST36)	No intervention
Gu (33)	China	AWGS 2019	25/27	77.68 ± 6.24/76.81 ± 5.78	Acupuncture (LI11, LI10, ST31, ST32, ST34, ST36, ST37, ST39, ST41) combined with exercise	Conventional treatment
Ling et al. (24)	China	AWGS 2019	21/21	64.7 ± 6.2/65.3 ± 5.8	Electroacupuncture (CV6, CV4, ST36, GB34, LU5, LI11, SP6, GV14, SP9, ST44, CV12, ST25, SP3, LR3, KI3) combined with exercise	Exercise
Liu et al. (34)	China	IWGS 2011	30/30	68.12 ± 5.84/70.17 ± 4.56	Weisanzhen Acupuncture (ST36, KI3, SP6) combined with exercise	Conventional treatment
Ma et al. (35)	China	AWGS 2014	30/30	65 ± 5.19/68 ± 7.41	Electroacupuncture (LI14, LI11, LI4, ST31, ST34, ST36, GB34, SP6) combined with nutritional support	Nutritional support
Pang (36)	China	AWGS 2019	30/30	70.2 ± 4.48/70 ± 4.75	Mediating acupuncture (ST36, KI3, SP6) combined with conventional treatment	Conventional treatment
Xin et al. (37)	China	AWGS 2014	23/25	70.35 ± 5.36/68.8 ± 5.08	Electroacupuncture (LI14, LI11, ST31, ST34) combined with nutritional support	Nutritional support
Yang et al. (38)	China	AWGS 2014	32/31	56 ± 8/57 ± 6	Warming needle moxibustion (LI14, LI11, LI4, ST31, ST34, ST36, GB34, SP6) combined with conventional treatment	Conventional treatment
Zhang et al. (23)	China	CECDTES 2021	47/47	72.84 ± 5.81/71.91 ± 6.03	Acupuncture (LI4, LI10, LI11, LI14, LI15, ST31, ST32, ST35, ST36, ST37, ST39, ST39, ST40, GB34, CV4, ST25, CV12) combined with conventional treatment	Conventional treatment

EG, Experimental group; CG, Control group. CECDTES, Chinese expert consensus on diagnosis and treatment for older adults with sarcopenia 2021, Conventional treatment: wellness education, nutritional support, daily activities or exercise.

### 3.3 Quality assessment

**Figure 2:**
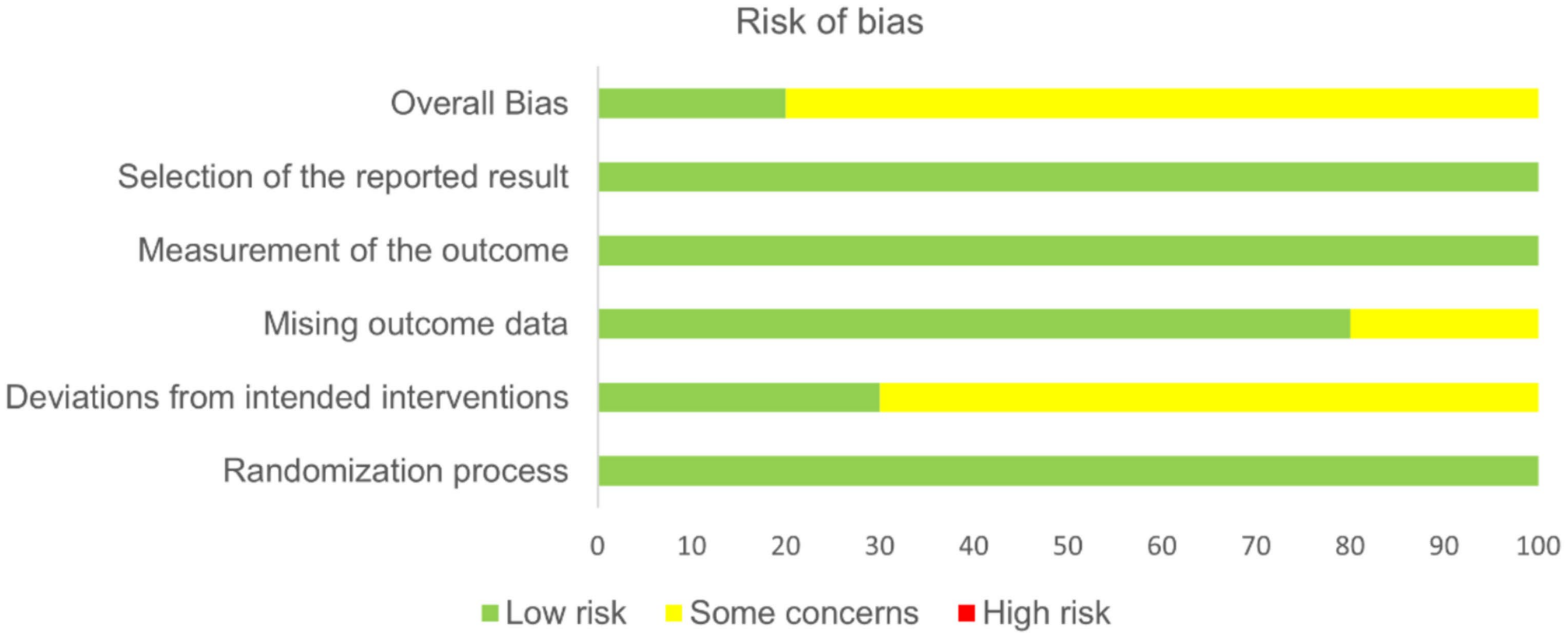
Summary of the RoB 2 quality assessment.

We used RoB 2.0 to evaluate the risk of bias in the 10 eligible studies. Our quality assessment considered six key criteria: (1) randomization process, (2) deviations from intended interventions, (3) missing outcome data, (4) outcome measurement, (5) selection of reported results, and (6) overall risk of bias. Figure 2 presents a summary plot of the risk of bias assessment, while Table 2 details the distribution of studies categorized as low risk, some concerns, or high risk of bias. All studies described the randomization process, outcome measurement, and selection of reported results. Three studies described the randomization method. Two studies described the missing outcome data. Overall, the assessment identified two studies with a low risk of bias, while the remaining studies exhibited some concerns. This reflects the challenges in conducting double-blind trials for complex interventions like acupuncture.

**Table 2:** Distribution of the RoB 2 graph.

Study ID	D1	D2	D3	D4	D5	Overall
Feng et al. (22)	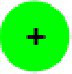	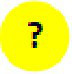	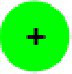	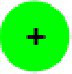	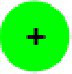	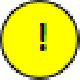
Soares Mendes Damasceno et al. (32)	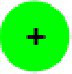	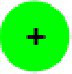	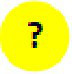	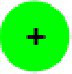	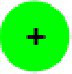	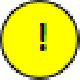
Gu (33)	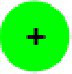	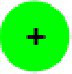	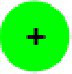	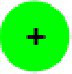	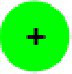	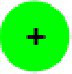
Ling et al. (24)	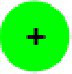	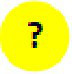	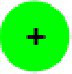	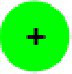	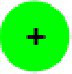	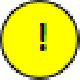
Liu et al. (34)	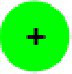	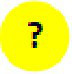	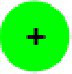	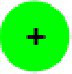	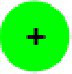	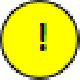
Ma et al. (35)	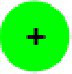	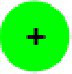	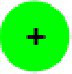	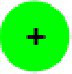	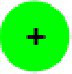	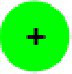
Pang (36)	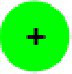	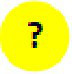	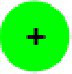	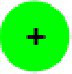	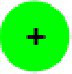	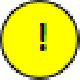
Xin et al. (37)	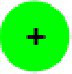	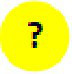	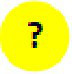	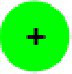	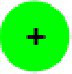	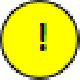
Yang et al. (38)	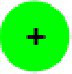	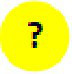	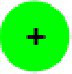	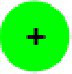	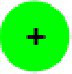	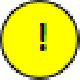
Zhang et al. (23)	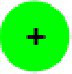	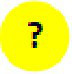	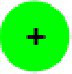	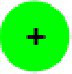	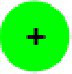	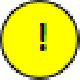

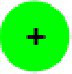
 Low risk; 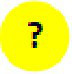 Some concerns; 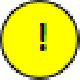 High risk. D1, Randomization process; D2, Deviations from intended interventions; D3, Missing outcome data; D4, Measurement of the outcome; and D5, Selection of the reported result.

### 3.4 Outcome measurements

This meta-analysis included only RCTs. The primary outcome measures assessed the effectiveness included total efficiency, muscle mass, grip strength, usual gait speed, the 6-min walk test, the short physical performance battery (SPPB) test, and C-reactive protein levels.

Among the 10 studies, the following outcomes were evaluated: total efficiency in 4 studies, muscle mass in 7 studies, grip strength in 7 studies, usual gait speed in 4 studies, 6-min walk test in 1 study, SPPB in 3 studies, C-reactive protein in 1 study. Due to the substantial heterogeneity among studies—arising from variations in sample size and study conditions—we applied a random-effects model for the meta-analysis.

#### 3.4.1 Total efficiency

**Figure 3:**
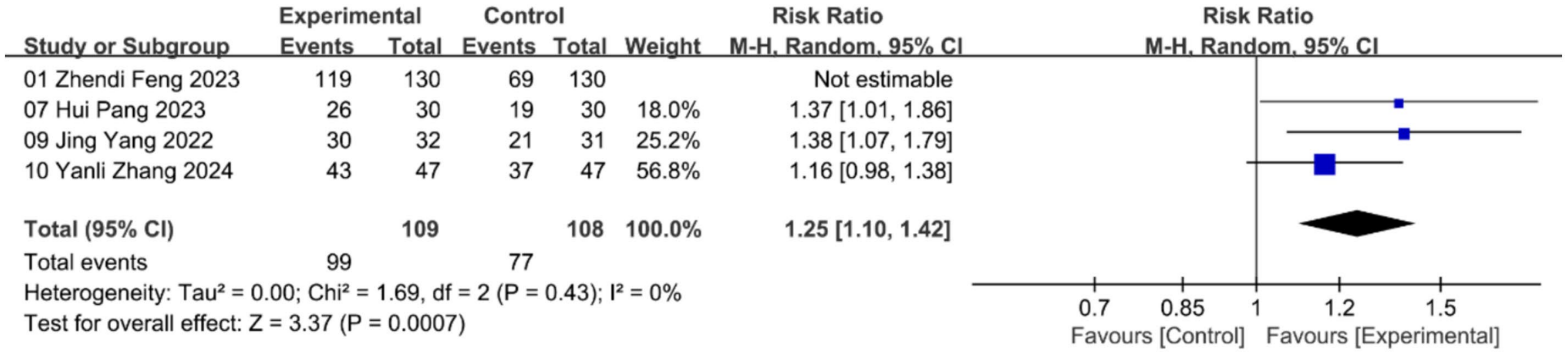
Forest plot of total efficiency with random-effects model.

Owing to variability in the types of outcome measures reported across trials, an intervention was classified as effective if it produced a significant improvement in the primary index defined within each study. The ratio of effective cases to the total number of cases was calculated as the total efficiency. A total of 4 studies assessed total efficiency. The meta-analysis using a random-effects model revealed that acupuncture significantly improved total efficiency compared to the control group, though with high heterogeneity (**n = 477; RR = 1.40, 95% CI: 1.13–1.73; heterogeneity: χ^2^ = 11.35, *p* = 0.01, I^2^ = 74%; Supplementary Figure S1). To address this heterogeneity, we conducted a sensitivity analysis. After excluding one study (22), the results using a random-effects model demonstrated statistically significant improvements with reduced heterogeneity (**n = 217; RR = 1.25, 95% CI: 1.10–1.42; heterogeneity: χ^2^ = 1.69, *p* = 0.43, I^2^ = 0%; Figure 3).

#### 3.4.2 Muscle mass

**Figure 4:**
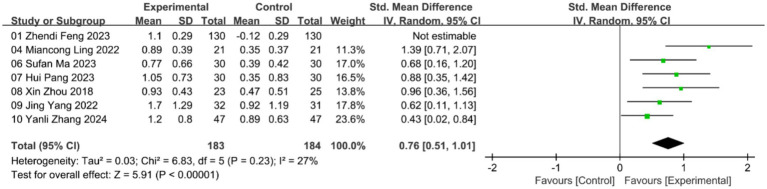
Forest plot of muscle mass with random-effects model.

A total of 7 studies assessed the effects of acupuncture on muscle mass. The meta-analysis using a random-effects model revealed a significant improvement in muscle mass compared to the control group, though with high heterogeneity (**n = 627; SMD = 1.31, 95% CI: 0.19–2.43; heterogeneity: χ^2^ = 200.93, *p* < 0.00001, I^2^ = 97%; Supplementary Figure S2). Due to the high heterogeneity, we conducted a sensitivity analysis. After excluding one study (22), the remaining five studies analyzed using a random-effects model showed statistically significant improvements with low heterogeneity (**n = 367; SMD = 0.76, 95% CI: 0.51–1.01; heterogeneity: χ^2^ = 6.83, *p* = 0.23, I^2^ = 27%; Figure 4).

#### 3.4.3 Grip strength

**Figure 5:**
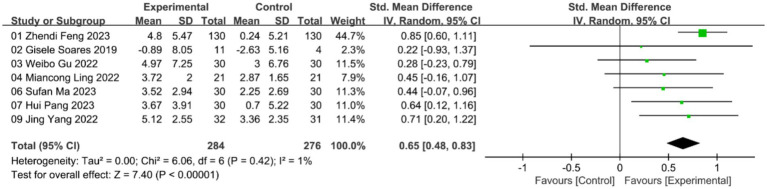
Forest plot of grip strength with random-effects model.

A total of 7 studies evaluated the effects of acupuncture on grip strength. The meta-analysis using a random-effects model revealed a significant improvement in grip strength compared to the control group, and with low heterogeneity (**n = 560; SMD = 0.65, 95% CI: 0.48–0.83; heterogeneity: χ^2^ = 6.06, *p* = 0.42, I^2^ = 1%; Figure 5).

#### 3.4.4 Usual gait speed

**Figure 6:**
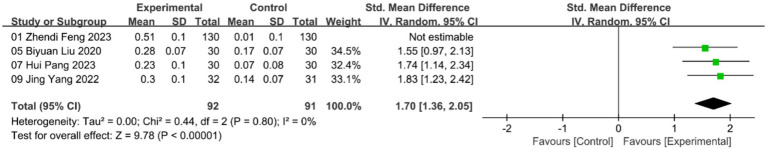
Forest plot of usual gait speed with random-effects model.

A total of 4 studies assessed the effects of acupuncture on usual gait speed. The meta-analysis using a random-effects model indicated a significant improvement in gait speed compared to the control group; however, high heterogeneity was observed (**n = 443; SMD = 2.53, 95% CI: 0.78–4.28; heterogeneity: χ^2^ = 114.59, *p* < 0.00001, I^2^ = 97%; Supplementary Figure S3). Given the high heterogeneity, we performed a sensitivity analysis. After excluding one study (22), the remaining five studies analyzed using a random-effects model showed statistically significant improvements with low heterogeneity (**n = 183; SMD = 1.70, 95% CI: 1.36–2.05; heterogeneity: χ^2^ = 0.44, *p* = 0.8, I^2^ = 0%; Figure 6).

#### 3.4.5 6-min walk test

A total of 1 study evaluated the effects of acupuncture on the 6-min walk test (6MWT). The meta-analysis using a random-effects model did not show statistically significant improvement compared to the control group (**n = 33; SMD = 0.64, 95% CI: −0.06–1.34; Supplementary Figure S4).

#### 3.4.6 Short physical performance battery

**Figure 7:**
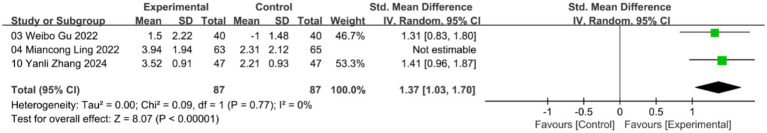
Forest plot of short physical performance battery with random-effects model.

Three studies examined the SPPB, and meta-analysis using a random-effects model showed that acupuncture led to significant improvement compared to the control group, though heterogeneity was high (*n* = 302, SMD = 1.15, 95% CI: 0.75 to 1.55; heterogeneity: χ^2^ = 5.27, *p* = 0.07, I^2^ = 62%; Supplementary Figure S5). To identify the source of heterogeneity, we conducted a sensitivity analysis. After excluding one studies (25), the random-effects model revealed a statistically significant improvement with lower heterogeneity (*n* = 174, SMD = 1.37, 95% CI: 1.03 to 1.70; heterogeneity: χ^2^ = 0.09, *p* = 0.77, I^2^ = 0%; Figure 7).

#### 3.4.7 C-reactive protein

One study investigated C-reactive protein levels, and meta-analysis using a random-effects model indicated a significant reduction with acupuncture compared to the control group (*n* = 94, SMD = −0.99, 95% CI: −1.42 to −0.56; Supplementary Figure S6).

### 3.5 Publication bias

We assessed publication bias for primary outcomes using Egger’s tests. The results suggest potential publication bias of muscle mass, yielding the following *p*-values: total efficiency (*p* = 0.208), muscle mass (*p* = 0.002), grip strength (*p* = 0.826), and usual gait speed (*p* = 0.715; Supplementary Figure S7–S10).

### 3.6 Evaluation of evidence quality

**Figure 8:**
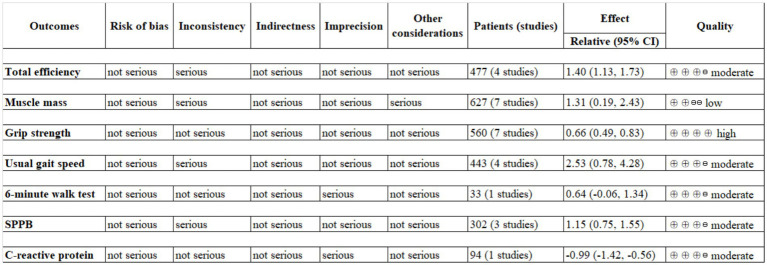
Evidence quality for study outcomes.

Figure 8 presents the overall certainty of evidence across studies. Based on the GRADE Working Group guidelines (32), the evidence quality varied across outcomes. Grip strength was rated as high-quality evidence, total efficiency, usual gait speed, 6-min walk test, SPPB and C-reactive protein were rated as moderate-quality evidence while muscle mass was supported by low-quality evidence. After assessing the studies using the GRADE framework, we found that the overall level of evidence was predominantly moderate. Several factors likely contributed to this outcome, including assessor blinding, concealed allocation, heterogeneity and sample size.

## 4 Discussion

Sarcopenia is typically managed through exercise therapy, nutritional supplementation (protein/vitamin D), and hormonal modulation (testosterone). However, these approaches have notable limitations, including side effects (e.g., cardiovascular risks), poor long-term adherence, and single-target mechanisms. Emerging biologics, such as myostatin antibodies, show potential in reversing muscle atrophy but remain expensive and lack comprehensive mechanistic validation (33).

This meta-analysis included 10 studies with a total of 754 patients and systematically evaluated the effects of acupuncture intervention on total efficiency, muscle mass, grip strength, usual gait speed, the 6-min walk test, SPPB, and C-reactive protein in sarcopenia patients. Studies exhibiting high heterogeneity underwent sensitivity and subgroup analyses to identify sources of variation. However, the results demonstrate that acupuncture intervention has significant potential to enhance total efficiency, muscle mass, grip strength, usual gait speed, and SPPB. Although only one study reported C-reactive protein, a significant potential of acupuncture was nevertheless observed. The significant heterogeneity observed is likely a reflection of the age of patients and the methodological diversity of acupuncture rather than statistical inconsistency. Due to substantial between-study heterogeneity, the present findings should be regarded as exploratory rather than definitive. Although the sensitivity analyses lent some support to the robustness of our findings, the uneven quality of the included RCTs necessitates a cautious interpretation of the results. Moreover, none of the included studies reported adverse effects associated with acupuncture. However, several studies did not explicitly report the absence of adverse events.

Research on the effects of acupuncture for improving sarcopenia-related indices is currently concentrated in animal rather than clinical studies. Electroacupuncture at ST36 has been shown to delay gastrocnemius atrophy in rats, potentially by upregulating the expression of Myod1, Myog, and Myh7 mRNAs, which control satellite cell differentiation and muscle fiber type transformation (34). However, these interventions show limited efficacy in improving the 6-min walk test (which primarily evaluates cardiopulmonary endurance) and the SPPB composite scores. This suggests that their effects are more focused on local muscle regeneration rather than enhancing overall systemic function. These characteristics highlight their clinical value in treating patients with atrophic sarcopenia.

For grip strength improvement, acupuncture acts via dual mechanisms: (1) reducing pro-inflammatory factors (TNF-*α*, C-reactive protein) to inhibit inflammation-driven muscle breakdown, and (2) stimulating spinal anterior horn motor neurons to enhance type I muscle fiber recruitment efficiency, improving neuromuscular coordination. Resistance exercise combined with nutritional supplementation (SMD = 0.95) suggests synergistic effects with acupuncture through metabolic regulation and mechanical loading.

Gait speed improvement reflects enhanced lower limb muscle function and reduced oxidative stress via mitochondrial respiratory optimization (e.g., Nrf2/HO-1 pathway activation to inhibit ferroptosis) (35).

C-reactive protein reduction underscores acupuncture’s systemic anti-inflammatory effects, mediated by vagal-adrenal axis activation to release norepinephrine, suppressing IL-6 and other pro-inflammatory cytokines. This aligns with warm acupuncture’s modulation of irisin and TNF-α, supporting metabolic-immune dual-pathway regulation (36)^.^

Total efficacy necessitates standardized protocols (acupoint combinations, stimulation parameters). Future studies should integrate multi-omics technologies to elucidate acupuncture’s regulatory networks on mitochondrial function (8), autophagy (37), and inflammation (38), validated through multicenter large-scale RCTs.

### 4.1 Highlights and limitations

This study represents the first meta-analysis to evaluate the efficacy of acupuncture in the treatment of sarcopenia. We evaluate recent research on the therapeutic effects of acupuncture intervention for sarcopenia. Given the absence of comprehensive reviews and discussions on acupuncture intervention in recent years, our work is both timely and necessary. This study systematically examines acupuncture intervention to assess the positive impact of acupuncture on sarcopenia. We offer valuable insights for future studies to identify optimal treatment approaches and provide reference evidence for translating preclinical findings into clinical applications. To date, exercise remains one of the most common and effective treatments for sarcopenia. However, our findings suggest that acupuncture interventions present a promising and effective form of alternative and complementary medicine. Clinical practice must adopt precision-based, combined intervention strategies. Current challenges include elucidating complex component-target interaction networks and establishing standardized frameworks for evaluating efficacy to optimize therapeutic outcomes.

This study has limitations. First, the heterogeneity observed across some studies likely arises from variations in intervention types, methods, frequency, duration, and the absence of standardized treatment protocols. Although this heterogeneity reflects the individualized approach of TCM syndrome differentiation and personalized treatment, it may hinder the generalizability and comparability of the findings. Second, the methodological quality of the included studies was suboptimal, with issues such as inadequate descriptions of allocation concealment and incomplete reporting of outcome data. The generally modest quality of the current evidence base underscores the necessity for future, more methodologically robust RCTs. Specifically, efforts should be made to improve blinding procedures and detailed reporting of randomization methods. Additionally, the small sample sizes and generally low quality of studies on multimodal combined interventions limit the strength of the evidence regarding the relationship between therapeutic effects and efficacy. The available evidence originates predominantly from studies conducted in specific regions; therefore, caution is warranted when generalizing these findings to populations of different ethnicities and cultural backgrounds. Future research should prioritize larger sample sizes and higher-quality multicenter trials to provide more robust evidence.

## 5 Conclusion

This study systematically evaluated the effects of acupuncture on sarcopenia and conducted a meta-analysis that demonstrated improvements in effectiveness, body composition, physical function, and blood biomarkers compared with the control group. Acupuncture demonstrated potential for enhancing physical function in sarcopenia patients. These findings provide significant scientific support for expanding treatment options for sarcopenia. Acupuncture, nutritional supplementation, and resistance exercise should be integrated into a multidimensional and inclusive therapeutic framework, particularly for individuals who respond poorly to or have low adherence to conventional protocols. Such approaches offer a promising avenue for addressing age-related disabilities in sarcopenia patients. For frail older adults who are unable to tolerate or adhere to high-intensity exercise, acupuncture offers a well-tolerated and easily implementable therapeutic option. In summary, the evidence presented in this study supports the integration of acupuncture into the clinical management pathway for sarcopenia. It represents not merely a therapeutic modality, but a holistic and individualized treatment paradigm tailored to the complexities of age-related disease. Future endeavors should focus on translating the potential of this ancient practice into unequivocal clinical guidelines within modern medicine through rigorously designed investigations.

## Data Availability

The original contributions presented in the study are included in the article/Supplementary material, further inquiries can be directed to the corresponding authors.
