# Supplementary material for: The potential of acupuncture in treating sarcopenia: a systematic review and meta-analysis of randomized controlled trials
Source: Front Public Health. 2025 Nov 10;13:1696030. doi: 10.3389/fpubh.2025.1696030 (PMC12640850; doi:10.3389/fpubh.2025.1696030)
Supplement: Supplementary file 1 [file Supplementary_file_1.zip › Supporting Information/0.Search data/sinomed.doc]

%0 Journal Article
%@ 1001-1528
%T 三子养亲汤加减联合针刺对慢性阻塞性肺疾病稳定期合并肌少症患者的临床疗效
%D 2024
%7 20240507
%K 三子养亲汤;针刺;常规治疗;慢性阻塞性肺疾病稳定期;肌少症
%V 46
%] 2024199348
%N 1
%J 中成药
%Q Clinical effects of Modified Sanzi Yangqin Decoction combined with acupuncture on patients with chronic obstructive pulmonary disease at stable stage complicated with sarcopenia
%X 目的:探讨三子养亲汤加减联合针刺对慢性阻塞性肺疾病稳定期合并肌少症患者的临床疗效。方法: 94例患者随机分为对照组和观察组,每组47例,对照组给予常规治疗,观察组在对照组基础上加用三子养亲汤加减联合循经针刺,疗程8周。检测临床疗效、中医证候评分、肺功能指标(FVC、FEV1、FEV1/FVC)、炎症因子(CRP、IL-6、TNF-α),肌肉特异性生物标志物(MSTN、IGF-1)、相关量表评分(SARC-F、SPPB)、骨骼肌质量指数变化。结果:观察组总有效率高于对照组(P<0.05)。治疗后。2组中医证候评分、炎症因子、MSTN、SARC-F评分降低(P<0.05),肺功能指标、IGF-1、SPPB评分、骨骼肌质量指数升高(P<0.05),以观察组更明显(P<0.05)。结论:三子养亲汤加减联合针刺对慢性阻塞性肺疾病稳定期合并肌少症患者临床疗效显著。
%A 张艳丽;宋昳星;徐丹
%+ (1)三亚市中医院呼吸科,海南三亚572000; (2)新疆医科大学附属中医医院针灸科,新疆乌鲁木齐830011; (3)新疆医科大学附属中医医院国家中医临床研究基地,新疆乌鲁木齐 830011
%& 107-111

%0 Journal Article
%@ 1009-8011
%T 循证精准护理联合八段锦运动对老年肌少症患者的应用效果及生活质量影响
%D 2024
%7 20240507
%K 循证精准护理;八段锦运动;肌少症;生活质量;应用效果
%V 42
%] 2024182338
%N 3
%J 中华养生保健·学术版
%X 目的:探讨循证精准护理联合八段锦运动对老年肌少症患者的应用效果及生活质量影响。方法:选取2021年6月-2023年1月江苏省中医院收治的80例老年肌少症患者作为研究对象,应用随机数表法将其分为观察组与对照组,每组40例。对照组采取常规护理,观察组在常规护理基础上增加循证精准护理联合八段锦运动,比较两组患者护理干预效果,肌肉衰减指标与运动功能,并比较两组患者干预前后的生活质量变化。结果:观察组护理干预总有效率明显高于对照组,差异有统计学意义(P<0.05);干预前两组患者握力、肌肉质量(Muscle mass,RASMI)比较,差异无统计学意义(P>0.05),干预后两组患者握力、RASMI均高于干预前,且观察组高于对照组,差异有统计学意义(P<0.05);干预前两组患者生活质量相关评分比较,差异无统计学意义(P>0.05),两组患者干预后生活质量相关评分均升高,且观察组高于对照组,差异有统计学意义(P<0.05)。结论:循证精准护理联合八段锦运动能够提升老年肌少症患者的干预效果,改善患者的运动能力,提升肌肉质量与握力,改善肌肉衰减情况,进一步提升患者生活质量。
%A 黄莹;戴卉;杨悦;陈茜;刘琳娜
%+ 江苏省中医院老年医学科,江苏南京 210000
%& 126-129

%0 Journal Article
%@ 1007-5615
%T 基于虚劳理论探析肝硬化肌少症
%D 2023
%7 20240507
%K 肝硬化肌少症;虚劳;因虚致损致劳;虚积互生;补虚祛瘀;治养结合;大黄蟅虫丸
%V 38
%] 2024162565
%N 6
%J 河北中医药学报
%Q Exploring Cirrhotic sarcopenia Based on Consumption Theory
%X 提要中医学的虚劳包含疾病范围广泛。现代医学的慢性消耗性疾病可纳入虚劳范畴。肌肉减少症是肝硬化的常见并发症,与肝硬化病人的生存率密切相关。肝硬化肌少症属于“虚劳”范畴,以脾肝肾三脏亏虚为本,瘀血痹阻为标,最终虚实夹杂,虚积互生,互为因果。故治疗宜补虚祛瘀、攻补兼施。临床处方用药宜立足于经典,灵活变通,以大黄蟅虫丸为主方化裁,治养结合,长期调摄。
%A 张瑜;苏春芝;张桐;孟宪鑫;张金丽;郑鑫淼;马雯
%+ (1)河北中医药大学研究生学院,石家庄050091; (2)河北中医药大学第一附属医院,石家庄 050011
%& 33-36

%0 Journal Article
%@ 1005-0957
%T 针刺治疗2型糖尿病并发肌少症的疗效观察
%D 2023
%7 20240507
%K 针刺疗法;针药并用;烧山火;2型糖尿病;肌少症
%V 42
%] 2023400583
%N 8
%J 上海针灸杂志
%Q Efficacy observation of acupuncture for type 2 diabetes complicated by sarcopenia
%X 目的:观察烧山火针刺治疗2型糖尿病并发肌少症的临床疗效。方法:将260例2型糖尿病并发肌少症的患者随机分为治疗组和对照组,每组130例。对照组根据2型糖尿病诊疗指南进行标准化治疗,治疗组在对照组治疗基础上予烧山火针刺治疗。观察两组治疗前后中医证候积分(各单项评分和总分)、肌少症相关观察指标(四肢骨骼肌含量、握力、6m步行速度和小腿围)、血糖指标(空腹血糖、餐后2h血糖和糖化血红蛋白的水平)和肝肾功能指标(血清谷丙转氨酶、尿素氮和肌酐的水平)的变化,比较两组临床疗效。结果:治疗组治疗后中医证候积分各单项评分和总分均低于同组治疗前(P<0.01),且均低于对照组治疗后(P<0.01)。治疗组治疗后肌少症相关观察指标和血糖指标均优于同组治疗前(P<0.01),且均优于对照组治疗后(P<0.01)。两组治疗后肝肾功能指标与同组治疗前比较,差异无统计学意义(P>0.05)。治疗组总有效率为91.5%,高于对照组的53.1%,差异具有统计学意义(P<0.05)。结论:在西药标准化治疗2型糖尿病的基础上采用烧山火针刺治疗可改善2型糖尿病并发肌少症的临床症状,有助于提高四肢骨骼肌含量和控制血糖,临床疗效优于单纯西药治疗。
%A 冯臻谛;梁静华;冯胜奎
%+ (1)中国中医科学院,北京 100091; (2)北京市西城区广外医院,北京 100055
%& 831-836

%0 Journal Article
%@ 1001-2001
%T 全身振动训练联合八段锦运动对老年肌少症患者姿势稳定性的影响
%D 2023
%7 20240507
%K 全身振动训练;八段锦;肌少症;姿势稳定性;跌倒
%V 38
%] 2023374479
%N 7
%J 中国康复
%X 目的:探讨全身振动训练(WBVT)联合八段锦运动对老年肌少症患者姿势稳定性的影响。方法:将51例老年肌少症患者随机分为对照组(n=18)、八段锦组(n=17)和联合治疗组(n=16)。3组患者均接受健康教育,八段锦组在健康教育基础上增加八段锦运动,联合治疗组在健康教育基础上先进行WBVT,随后进行八段锦运动。分别于治疗前、治疗12周后采用“起立-行走”计时测试(TUGT)、5次坐站计时测试(FTSST)、修订版跌倒效能量表(MFES)和Pro-Kin 254动静态平衡测试系统评估3组患者的姿势稳定性和跌倒恐惧程度。结果:治疗12周后,八段锦组和联合治疗组TUGT、FTSST、MFES和稳定及限值(LOS)均增加(均P<0.05);运动长度、运动椭圆面积、总偏移指数均减少(均P<0.05);联合治疗组TUGT、FTSST、运动长度、运动椭圆面积、总偏移指数均优于八段锦组和对照组水平(均P<0.05),MFES和LOS均高于八段锦组和对照组(均P<0.05);而八段锦组上述指标亦显著优于对照组(均P<0.05)。结论:WBVT联合八段锦运动能有效改善老年肌少症者姿势控制能力,提高动态和静态姿势稳定性,从而降低跌倒风险,该联合治疗方案值得在临床上推广与应用。
%A 黄墩兵;林忠华;姜财;贾小飞;宋薇;柯晓华
%+ (1)同济大学附属上海市第四人民医院康复医学科,上海 200434; (2)福建省立医院康复二科,福州 350001; (3)宁夏回族自治区人民医院康复医学科,银川 750002
%& 430-433

%0 Journal Article
%@ 1005-0698
%T 参苓白术散治疗老年男性肌少症的成本-效用分析
%D 2023
%7 20240507
%K 参苓白术散;肌少症;成本-效用分析;药物经济学
%V 32
%] 2023324998
%N 4
%J 药物流行病学杂志
%Q Cost-utility analysis of Shenling Baizhu powder in treatment of sarcopenia in older men
%X 目的:评价参苓白术散治疗老年男性肌少症的成本-效用。方法:280例老年男性肌少症患者随机分为干预组和对照组各140例,两组患者均给予营养运动治疗,干预组在营养运动治疗基础上加用参苓白术散,两组患者均随访24周。从卫生体系角度出发,利用TreeAge Pro 2011软件建立决策树模型,对两种治疗方案进行成本-效用分析,并采用敏感性分析对结果的不确定性进行评价。结果:与对照组相比,干预组方案成本(5019.46元vs.4864.57元)、效用[0.90357质量调整生命年(QALYs)vs.0.89430 QALYs]均较高,增量成本-效用比为16712.05元/QALY,低于意愿支付阈值。敏感性分析结果与成本-效用分析结果一致。结论:与单独应用营养运动治疗相比,老年男性肌少症患者应用参苓白术散联合营养运动治疗更具有成本-效用。
%A 蒋欢欢;申慧琴;王志秀;陈云飞;郭晖;徐娜
%+ (1)华北理工大学附属医院药学部,河北唐山 063000; (2)河北北方学院附属第二医院药剂科,河北张家口 075000; (3)河北北方学院附属第二医院临床营养科,河北张家口 075000
%& 376-383

%0 Journal Article
%@ 1673-6273
%T 四物汤在卵巢衰老大鼠骨骼肌中的雌激素样作用机制研究
%D 2023
%7 20240507
%K 四物汤;肌肉减少症;IGF-1/PI3K/AKT/mTOR;4-乙烯基环己烯二环氧化合物;植物雌激素
%V 23
%] 2023235731
%N 4
%J 现代生物医学进展
%Q Study on the Estrogen-like Action Mechanism of Siwu Decoctionin Skeletal Muscle of Ovarian Aging Rats
%X 目的:探讨四物汤通过胰岛素样生长因子-1(Insulin-ike growth factors-1,IGF-1)/磷脂酰肌醇3-激酶(PI3K)/蛋白激酶B(AKT)/雷帕霉素靶蛋白(Mammalian target of rapamycin,mTOR)mTOR信号通路发挥对4-乙烯基环己烯二环氧化合物(4-Vinylcyclohexene Diepoxide,VCD)诱导的卵巢衰老大鼠骨骼肌的保护作用及其分子机制。方法:选用28日龄雌性F-344大鼠,随机选取6只作为空白对照组,剩余大鼠连续腹腔注射VCD溶液(160mg/kg/d)20天,每天阴道涂片检测动情周期,连续观察12d无角化细胞或仅有少量角化细胞即符合"卵巢衰老"表现,经动情周期筛选出24只造模成功的大鼠,分为模型组(Model)、阳性(戊酸雌二醇,E2)对照组、四物汤高剂量(SWT-H)组和四物汤低剂量(SWT-L)组,每组6只,灌胃持续3周后取材。取大鼠骨骼肌组织进行HE染色观察病理组织结构;MASSON染色骨骼肌纤维形态变化;RT-PCR检测骨骼肌组织中各基因的mRNA水平。结果:与空白组比较,模型组骨骼肌肌纤维排列疏松,分布不均且有断点,胞浆不均,细胞核增多,出现增生的结缔组织,胶原纤维逐渐增多且肌纤维间距变宽;与模型组相比,E2组、SWT-H组与SWT-L组肌纤维排列相对整齐,胞浆较为均匀。肌肉形态较规则,纤维间距缩短,胶原纤维减少。RT-PCR结果显示,与空白组相比,模型组IGF-1、PI3K、AKT、mTOR基因的mRNA表达量明显下调,E2组、SWT-H组与SWT-L组的IGF-1、PI3K、mTOR的表达明显上升,SWT-H组AKT表达无明显变化,无统计学意义,SWT-L组AKT表达相对降低。与模型组相比,E2组、SWT-H组与SWT-L组的IGF-1、AKT、mTOR的表达明显增加,且具有剂量依赖性,PI3K表达增加,但无明显剂量依赖性。结论:四物汤可以明显改善VCD诱导的卵巢衰老大鼠的肌肉减少情况,其机制可能是通过IGF-1-PI3K-AKT-mTOR信号通路来发挥其抑制肌肉流失的作用。
%A 杨佳迪;石丹宁;陈瑶;何悦双;赵丕文
%+ 北京中医药大学生命科学学院,北京 100029
%& 607-612

%0 Journal Article
%@ 1672-7134
%T 自拟增肌消渴汤联合针刺治疗糖尿病合并肌少症临床观察
%D 2023
%7 20240507
%K 糖尿病;肌少症;健脾益肾;针药联用;丙辰针法
%V 35
%] 2023228468
%N 3
%J 中医药临床杂志
%Q Clinical Observation of Self-enhancing Muscle Thirst Soup Combined with Acupuncture for Diabetes and Sarcopenia
%X 目的:观察增肌消渴汤联合丙辰针法治疗糖尿病肌少症(diabetic sarcopenia)的临床疗效。方法:选取符合标准的糖尿病肌少症患者60例,按照随机对照的原则将患者分为治疗组和对照组。对照组采取抗阻训练,治疗组在抗阻训练基础上,加用增肌消渴汤,并以丙辰针法(取穴阳明经并据《灵枢》阴阳系日月篇丙主左手阳明,辰主左足阳明,提插补之)行针刺治疗,原则上均不变动原有降糖方案。观察2组临床疗效的差异,分析组方药物及功效配伍。结果:治疗组总有效率90.00%,对照组总有效率63.33%,差异有统计学意义(P<0.05);2组治疗后FPG、HbA1c较治疗前改善(P<0.05);2组治疗后握力、步速较治疗前提高(P<0.05);治疗组治疗后FPG和HbA1c水平低于对照组(P<0.05);治疗组治疗后握力、步速水平高于对照组(P<0.05)。结论:增肌消渴汤联合丙辰针法治疗糖尿病肌少症临床效果显著,患者受益良多,可有效改善肌力,提高握力与步速,具有良好的临床应用前景。
%A 刘佳青;徐筱玮;唐琳;王越;徐彤;曲传鑫
%+ (1)山东中医药大学第一临床医学院,山东济南 250000; (2)青岛市中医医院,山东青岛 266000; (3)青岛大学附属医院,山东青岛 266000
%& 555-559

%0 Journal Article
%@ 1001-1242
%T 易筋经在骨骼肌减少症中的潜在作用
%D 2023
%7 20240210
%V 38
%] 2024149022
%N 12
%J 中国康复医学杂志
%X 截止2020年底,我国老年人口数量位居全球榜首,人口老龄化进程的不断加剧造成了骨骼肌减少症(肌少症,sarcopenia)患病率的攀升。据推测,全球目前约有5000万人罹患肌少症,预计至2050年,患病人数将高达5亿^([1])。肌少症起病隐匿,常表现为增龄性的肌纤维萎缩,肌肉数量、力量下降,易诱发患者平衡等功能障碍,增加老年人跌倒、骨折甚至死亡的风险,严重威胁老年人的生活质量。[第一段]
%A 唐强;穆姿辰;朱路文;李宏玉;杨善军;李保龙;李慧欣;赵晓倩;尹侠
%+ (1)黑龙江中医药大学附属第二医院,黑龙江省哈尔滨市150001; (2)黑龙江中医药大学; (3)上海傅利叶智能科技有限公司; (4)北京中医药大学
%& 1757-1761

%0 Journal Article
%@ 1001-2001
%T 太极拳对老年肌少症的影响
%D 2023
%7 20240210
%V 38
%] 2024128269
%N 11
%J 中国康复
%Q Effects of 12 Weeks of Tai Chi on Neuromuscular Responses and Postural Control inElderly Patients with Sarcopenia:A Randomized,Controlled Trial
%X 肌少症是一种以随着年龄增长发生的肌肉质量和力量下降为特征的疾病。这项研究评估了为期12周的太极拳训练对患有肌少症的老年患者的神经肌肉反应和改善姿势控制的疗效。受试者为60名患有肌少症的老年患者,随机分为太极拳组和对照组。两组均接受每两周一次的健康教育课程,持续12周,而太极拳组额外加入了每周进行3次,每次40分钟的太极拳课程,持续12周。在基础课程和太极拳课程结束后,使用表面肌电图评估下肢肌肉的神经肌肉反应时间。[第一段]
%A 徐岱松[译]
%+ 不详
%& 663

%0 Journal Article
%@ 0255-2930
%T 电针阳明经腧穴治疗肌少症:随机对照试验
%D 2023
%7 20240210
%K 肌少症;电针;骨骼肌质量;治痿独取阳明;随机对照试验
%V 43
%] 2023477094
%N 10
%J 中国针灸
%Q Electroacupuncture at acupoints of yangming meridians for sarcopenia:a randomized controlled trial
%X 目的:观察电针阳明经腧穴治疗肌少症的临床疗效。方法:将60例肌少症患者随机分为观察组和对照组,每组30例。对照组患者予肌少症常规营养疗法。观察组在对照组基础上针刺双侧臂臑、曲池、足三里、阳陵泉等穴,同侧曲池、足三里连接电针,予断续波,频率2 Hz,电流强度1－10 mA,每周2次,每次间隔3d。两组均治疗12周。分别于治疗前后观察两组患者四肢骨骼肌质量指数(ASMI)、握力、6m步行时间、体脂率及体水分率。结果:治疗后,两组患者ASMI及握力较治疗前提高、6m步行时间较治疗前缩短(P<0.05),观察组患者体脂率较治疗前降低、体水分率较治疗前升高(P<0.05);观察组患者ASMI、握力、体水分率高于对照组(P<0.05),6m步行时间短于对照组、体脂率低于对照组(P<0.05)。结论:电针阳明经腧穴可有效改善肌少症患者骨骼肌质量、肌肉功能、体脂率及体水分率,使肌肉、脂肪分布更趋合理。
%A 马素凡;吕万勇;朱茜扬;李慧佳;李晶晶;时倩;鲁欣儒;姚涵月;张彩荣
%+ (1)南京中医药大学附属南京中医院针灸科,江苏南京210022; (2)江苏省老年学学会老年营养专业委员会
%& 1114-1117

%0 Journal Article
%@ 1002-6975
%T 健身气功五禽戏在透析患者肌少症运动康复中的应用研究
%D 2023
%7 20240210
%K 五禽戏;透析;肌少症;运动康复;中医护理
%V 38
%] 2023474468
%N 20
%J 护士进修杂志
%Q Application of fitness qigong wuqinxi in exercise rehabilitation of dialysis patients with sarcopenia
%X 目的:探讨健身气功五禽戏对透析患者肌少症骨骼肌肌肉质量、力量、功能、人体测量指标及疲乏的影响。方法:便利抽样法抽取某三甲医院肾内科透析住院患者80例作为研究对象,按照随机信封法分为对照组和观察组,每组各40例。对照组采用常规治疗护理措施,观察组在对照组常规治疗护理措施基础上进行健身气功五禽戏锻炼。比较2组患者干预前、干预12周后骨骼肌质量指数、握力、体重指数、三角肌皮褶厚度、中臂围、中臂肌围、6m步速和简易躯体功能量表得分、疲乏程度的差异。结果:观察组干预12周后握力、三角肌皮褶厚度、中臂围、中臂肌围、6m步速和简易躯体功能量表评分均高于对照组(P<0.05);疲乏得分低于对照组(P<0.05);骨骼肌质量指数、体重指数与对照组对比,差异无统计学意义(P>0.05)。结论:健身气功五禽戏可改善透析患者肌少症骨骼肌肌肉力量、人体测量指标、肌肉功能,减轻疲乏程度。
%A 郭月月;薄祥敏;刘胜凤;殷玲;祝玲;赵如琴
%+ 南京中医药大学附属医院江苏省中医院,江苏南京 210029
%& 1865-1869

%0 Journal Article
%@ 1008-1879
%T 刘军教授治疗肌少症的学术经验撷萃
%D 2023
%7 20240210
%K 肌少症;针刀疗法;穴位注射;推拿按摩;辨证施治
%V 14
%] 2023448187
%N 12
%J 按摩与康复医学
%X 刘军教授临证20余年,活用经典古方,学贯中西,践行大医精诚,展现了医者、师者的人格魅力,热心给病患解决疾苦,更将所学、所思、所用毫无保留地传授给学生,笔者长期跟师侍诊,耳闻目濡,现将老师治疗肌少症的学术经验粗略总结,供同道分享。
%A 董云鹏;吕朝晖;李伟举;曾令烽;刘军
%+ (1)广州中医药大学第五临床医学院(广东省第二中医院),广东广州510095; (2)广州中医药大学第二临床医学院(广东省中医院),广东广州510120; (3)广东省中医药科学院,骨与关节退变及损伤研究团队,广东广州 510120
%& 29-31,34

%0 Journal Article
%@ 1005-0957
%T 温针灸治疗维持性血液透析患者肌少症的疗效观察及对血清irisin和TNF-α的影响
%D 2022
%7 20230725
%K 针灸疗法;温针疗法;肌少症;肾透析;血液透析
%V 41
%] 2023145872
%N 12
%J 上海针灸杂志
%Q Effects of Needle Warming Therapy on Sarcopenia in Maintenance Hemodialysis Patients and Its Effect on Serum Irisin and TNF-α
%X 目的:观察温针灸治疗维持性血液透析患者肌少症的临床疗效及其对患者血清鸢尾素(irisin)和肿瘤坏死因子-α(TNF-α)的影响。方法:将66例患者随机分为对照组和观察组,每组33例。对照组予常规治疗,观察组在此基础上予温针灸治疗。比较两组治疗前后四肢骨骼肌质量指数(ASMI)、握力、步速、简明健康状况调查表(SF-36)评分和日常生活能力(ADL)评分的变化,比较两组治疗前后血清irisin和TNF-α水平,比较两组临床疗效。结果:观察组总有效率高于对照组(P<0.05)。治疗后,两组ASMI、握力和步速均较治疗前提高(P<0.05),且观察组均优于对照组(P<0.05);两组SF-36和ADL评分均升高(P<0.05),且观察组评分均高于对照组(P<0.05)。对照组治疗后血清irisin水平升高(P<0.05),血清TNF-α水平无明显变化(P>0.05);观察组治疗后血清irisin水平升高(P<0.05)、血清TNF-α水平降低(P<0.05),且均优于对照组(P<0.05)。结论:在常规治疗基础上,温针灸可增加维持性血液透析肌少症患者的骨骼肌质量,增强肌力,改善肌肉功能,提高患者生存质量及生活自理能力,可能与其调节血清irisin和TNF-α水平有关。
%A 杨靖;黄英;李娟
%+ 北京中医医院怀柔医院,北京 101400
%& 1195-1199

%0 Journal Article
%@ 1000-338X
%T 八段锦联合抗阻运动干预老年肌少症30例
%D 2022
%7 20230725
%K 肌少症;八段锦;抗阻运动;肌力;日常生活能力
%V 53
%] 2023129360
%N 11
%J 福建中医药
%Q Baduanjin Combined with Resistive Exercise Treatment of Elderly Patients with Sarcopenia in 30 Cases
%X 目的:探讨八段锦联合抗阻运动对老年肌少症患者肌力及日常生活能力的影响。方法:选取老年肌少症患者60例,采用随机数字表法分为对照组31例和观察组32例。治疗期间对照组脱落1例,观察组脱落2例。对照组采用常规基础病治疗与护理,观察组在对照组干预基础上配合八段锦联合抗阻运动干预,每周干预3次,2组均干预12周。比较2组干预前后手握力、5次起坐试验时间、日常步行速度及改良Barthel指数(MBI)变化情况。结果:与治疗前比较,2组治疗后手握力、日常步行速度、MBI评分均明显提高(P<0.05),5次起坐试验时间明显减少(P<0.05);与对照组比较,观察组治疗后手握力、日常步行速度、MBI评分均明显提高(P<0.05),5次起坐试验时间明显减少(P<0.05)。结论:八段锦联合抗阻运动可以改善老年肌少症患者的肌力,提高其日常生活能力,值得在社区老年人群中推广应用。
%A 许雪琛;郭恒群;林健
%+ 福州市长乐区第二医院,福建福州 350211
%& 60-62

%0 Journal Article
%@ 1007-1334
%T 中医药治疗肌少症的临床应用及治疗机制初探
%D 2022
%7 20230725
%K 肌少症;痿证;中医药疗法;临床应用;作用机制;研究进展
%V 56
%] 2023122919
%N 12
%J 上海中医药杂志
%Q A preliminary investigation on clinical application and therapeutic mechanism of traditional Chinese medicine in treatment of sarcopenia
%X 肌少症被定义为老年人骨骼肌质量、力量和功能的进行性下降,伴随着身体虚弱、功能残疾、跌倒、住院和死亡,并且由于预期寿命的增加和全球老年人口的增长,本病正在成为一种主要的老年疾病。中医药疗法治疗肌少症具有独特优势。基于传统中医理论及国内外与肌少症相关的文献研究发现,采用中药、功法、针刺、推拿等治疗肌少症取得了一定的进展,但具体机制研究相对较少。总体而言,中医药对肌少症的研究还处于早期阶段,还需要开展高质量临床循证研究及基础研究,以提升中医药在肌少症防治中的作用。
%A 张禹杰;方淑蓓;岑俊;元唯安
%+ (1)上海中医药大学附属曙光医院,上海 201203; (2)上海市生物医药科技发展中心,上海 201203; (3)上海建工医院,上海 200083
%& 16-22

%0 Journal Article
%@ 0254-1769
%T 11种运动对老年肌少症患者身体功能改善效果的网状Meta分析
%D 2022
%7 20230725
%K 老年肌肉减少症;运动;身体功能;肌肉质量;肌肉力量;护理
%V 57
%] 2023104606
%N 21
%J 中华护理杂志
%Q The improvement effect of 11 kinds of exercise protocols on elderly patients with sarcopenia:a network Meta analysis
%X 目的:通过网状Meta分析评价11种运动训练对老年肌少症患者身体功能、肌肉力量和肌肉质量改善效果的影响。方法:检索Web of Science、PubMed、Embase、Cochrane Library、CINAHL、中国知网、中国生物医学文献数据库、万方数据库和维普数据库中关于运动训练对老年肌少症患者身体功能、肌肉力量和肌肉质量改善效果的随机对照试验。检索时限为建库至2022年1月。采用Stata 15.0软件进行网状Meta分析。结果:纳入34项研究,共2199例患者。网状Meta分析结果表明,与非运动对照组相比,阻力训练、小组综合训练、八段锦、全身振动训练、家庭综合训练可改善老年肌少症患者的身体功能(P<0.05);阻力训练和壶铃训练可改善老年肌少症患者的握力(P<0.05);阻力训练可改善老年肌少症患者的骨骼肌指数(P<0.05)。结论:在改善老年肌少症患者身体功能、肌肉力量和质量方面,阻力训练是目前最优的运动干预措施,其次为小组综合训练和全身振动训练,但仍需更多研究进一步论证。
%A 王丽丽;田丽雅;牛琪;柳韦华
%+ 山东第一医科大学护理学院,泰安市 271000
%& 2652-2660

%0 Journal Article
%@ 2095-8757
%T 太极拳锻炼对老年慢性肾脏病并发肌少症患者的功能改善作用
%D 2022
%7 20230518
%K 慢性肾脏病;肌少症;老年人;运动平衡功能;功能性步态评价;肾功能
%V 9
%] 2022500660
%N 2
%J 中华老年病研究电子杂志
%Q To explore the effect of Taijiquan exercise on the improvement of motor and balance function and renal function in elderly patients with chronic kidney disease complicated with sarcopenia
%X 目的:探讨太极拳锻炼对老年慢性肾脏病并发肌少症患者运动和平衡功能的改善作用及对肾功能的影响。方法:选取2018年12月至2020年5月在浙江医院就诊的73例老年慢性肾脏病并发肌少症患者为研究对象。其中36例给予常规内科治疗(对照组),37例在此基础上给予太极拳锻炼干预12周(观察组)。比较干预前后两组患者四肢骨骼肌质量指数(ASMI)、握力指数、6m步行速度、功能性步态评价(FGA)评分、血肌酐、估算的肾小球滤过率(eGFR)以及尿微量白蛋白等指标。计量资料的组间比较采用t检验,计数资料的比较采用χ^(2)检验。结果:干预12周后,观察组患者ASMI、握力指数、6米步行速度、FGA评分较干预前均有明显提升,且明显高于对照组患者(t=2.876、4.375、4.982、4.582,P<0.01)。干预前后,观察组患者仅尿微量白蛋白水平的差异有统计学意义(F=5.396,P<0.05),其中干预12周后的尿微量白蛋白水平明显低于干预前和首次干预24h后(P<0.01)。干预12周后,观察组患者尿微量白蛋白水平明显低于对照组患者(t=2.557,P<0.01)。结论:太极拳运动能够提高老年慢性肾脏病并发肌少症患者的肌肉质量,改善平衡功能,保护肾功能,提高生活质量。
%A 陶有娣;黄雄昂;林坚;汤洋
%+ 浙江医院康复医学科,杭州 310013
%& 18-21

%0 Journal Article
%@ 1006-9771
%T 慢性病和功能障碍老年人身体活动的健康效果:系统综述的系统综述
%D 2022
%7 20230518
%K 老年人;慢性病;功能障碍;身体活动;锻炼;康复;系统综述
%V 28
%] 2022456337
%N 9
%J 中国康复理论与实践
%Q Effect of physical activity on health conditions and functioning for aging people with chronic diseases and functioning:a systematic review of systematic reviews
%X 目的:基于世界卫生组织国际分类家族(WHO-FICs)构建身体活动对慢性病和功能障碍老年人健康及健康相关状况的干预效果范畴及架构。方法:运用《国际疾病分类第十一次修订本》(ICD-11)和《国际功能、残疾和健康分类》(ICF)对老年人的健康及健康状况进行系统分析。检索PubMed、CBM、Scopus、Web of Science、Embase、中国知网、维普、万方等数据库,收集建库至2022年8月8日关于身体活动干预对慢性病和功能障碍老年人健康及健康相关状况康复效果的系统综述进行综述。结果:最终纳入系统综述11篇,来自6个国家,共涉及316项相关随机对照试验和28 169名参与者,主要包括老龄化、公共健康、康复、身体锻炼等领域。与老年人健康状况相关的疾病主要集中于痴呆、帕金森病、精神分裂症、抑郁症、认知障碍,骨质疏松症、关节炎、肌少症,慢性心力衰竭、冠心病、高血压、脑卒中,2型糖尿病,慢性肺病。干预方案中,活动形式有平衡、功能和耐力训练、灵活性训练,步行、伸展、上下肢训练、瑜伽、气功、太极拳等,活动频率每周1－7次,活动强度为中度,活动持续时间4周－10年。健康结局涉及降低疾病风险,促进整体认知功能、精神功能、肌肉耐力、心肺耐力、柔韧性等的改善和发展,改善步行、移动、平衡、灵活性、姿势控制等基本动作技能,改善睡眠质量,缓解焦虑,减少压力,增加社会参与和人际互动交流,提升幸福感等。结论:基于WHO-FICs构建了慢性病和功能障碍老年人参与身体活动产生的健康效果的理论架构。老龄化导致的健康及健康相关状况主要涉及认知、心理和精神系统,骨骼肌肉关节系统,心血管系统,代谢系统,呼吸系统。身体活动的干预方案可以分为体适能类、技能类和运动项目类。健康和功能结局主要表现在降低慢性病的发病率,改善功能,提高身体活动水平,促进心理健康,提高生活质量和福祉。
%A 袁媛;杨剑
%+ (1)华东师范大学/青少年健康评价与运动干预教育部重点实验室,上海市 200241; (2)华东师范大学体育与健康学院,上海市 200241
%& 1003-1011

%0 Journal Article
%@ 1008-1879
%T 基于脾主肌肉探讨肌少症的电针治疗
%D 2022
%7 20230518
%K 电针;肌少症;脾主肌肉
%V 13
%] 2022406515
%N 17
%J 按摩与康复医学
%Q Study on Electroacupuncture Treatment of Sarcopenia Based on Spleen Governing Muscle
%X 肌少症是一种与增龄相关的综合退行性病征,主要表现为肌肉数量与质量的下降及功能的减退。本文基于“脾主肌肉”理论内涵,认为脾的生理功能与肌少症诊断的三大要素密切相关,笔者通过长期的跟师学习,总结导师治疗肌少症的经验方法,从脾入手,以中脘、天枢、气海为主要穴位处方,探讨电针治疗肌少症的可靠性和有效性,为今后防治肌少症提供更多的临床依据。
%A 马素凡;吕万勇;张彩荣
%+ (1)南京中医药大学附属南京中医院,江苏南京 210001; (2)南京中医药大学,江苏南京 210029; (3)江苏省老年学学会老年营养专业委员会,江苏南京 210000
%& 10-13,17

%0 Journal Article
%@ 1008-1879
%T 新编易筋经锻炼对老年肌少症患者下肢运动功能和平衡功能的影响
%D 2022
%7 20230518
%K 肌肉减少症;老年患者;新编易筋经;运动功能;平衡功能;步行速度
%V 13
%] 2022406512
%N 17
%J 按摩与康复医学
%Q Effect of New Yijinjing Exercise on Lower Limb Motor Function and Balance Function in Senile Patients with Sarcopenia
%X 目的:探讨新编易筋经锻炼治疗老年肌少症患者的临床疗效,以及对患者下肢运动功能和平衡功能的影响。方法:将80例老年肌少症患者随机分为练功组和对照组各40例,在基础治疗上,练功组予以新编易筋经锻炼,对照组予以运动疗法训练,治疗前后分别评估患者Fugl-Meyer下肢运动功能评分(FMA)、Berg平衡量表(BBS)和步速,并比较治疗后两组疗效差异。结果:治疗期间,由于患者个人家庭原因,练功组有1例、对照组有2例中途主动要求退出,其余患者均顺利完成治疗。治疗1个疗程后,两组老年肌少症患者总体疗效、FMA、BBS和步速评分均有改善,练功组疗效显著优于对照组(P<0.05)。结论:新编易筋经作为传统健身功法之一,能有效改善老年肌少症患者下肢运动功能和平衡功能,值得临床和社区推广运用。
%A 彭天忠;朱满华;林星镇;袁娟;周峰;胡思彦;侯新聚
%+ 南昌市洪都中医院,江西南昌 330008
%& 21-26

%0 Journal Article
%@ 1671-7813
%T 易筋经功法治疗骨伤科疾病的研究进展
%D 2022
%7 20230120
%K 易筋经;颈椎病;腰痛病;膝骨关节炎;冻结肩;肌少症
%V 36
%] 2022366780
%N 4
%J 实用中医内科杂志
%Q Research Progress of Yijin jing(易筋经)in the Treatment of Orthopedic Diseases
%X 易筋经是我国传统导引术之一,经过漫长的历史演变与传承,逐渐形成了以“易筋经十二势”为核心内容的理论体系。长期坚持易筋经功法训练可以达到防治疾病、延年益寿的效果。文章总结了近年易筋经功法在治疗骨伤科疾病的相关临床研究,包括颈椎病、腰痛病、膝骨关节炎、冻结肩、肌少症等骨伤科常见疾病,其临床疗效显著,简单易学,老少皆宜,易于临床推广,富有创新性。
%A 吕博松;邰东旭
%+ (1)辽宁中医药大学,辽宁沈阳 110847; (2)辽宁中医药大学附属医院,辽宁沈阳 110032
%& 94-96

%0 Journal Article
%@ 1672-8351
%T 补中益气汤加减辅助治疗老年肌少症的临床疗效观察
%D 2021
%7 20230120
%K 老年肌少症;补中益气汤;常规治疗;Barthel评分;C反应蛋白
%V 18
%] 2022339529
%N 11
%J 北方药学
%X 目的:探讨老年肌少症患者应用补中益气汤加减辅助治疗的效果。方法:选择我院收治的80例老年肌少症患者,以随机数字表法将其分为研究组(40例,常规治疗)与对照组(40例,常规治疗+补中益气汤加减辅助治疗),对比其疗效。结果:研究组患者治疗总有效率(95.0%)显著高于对照组(75.0%),治疗后肌肉质量、肌肉力量、肌肉功能也高于对照组,治疗后Barthel评分、各项生活质量评分高于对照组,治疗后C反应蛋白水平低于对照组,以上均有统计学差异(P<0.05)。结论:老年肌少症患者采取补中益气汤加减治疗可取得很好的疗效,还能显著提升其日常生活能力和生活质量。
%A 吕罗岩;陈向民;王建伟;苏行;申晓华
%+ 蚌埠市第一人民医院,安徽蚌埠 233000
%& 29-31

%0 Journal Article
%@ 1672-951X
%T 四君子汤加味联合弹力带训练治疗老年肌少症脾胃气虚证的疗效观察
%D 2022
%7 20230120
%K 肌少症;老年;脾胃气虚证;四君子汤加味;弹力带训练;肌肉质量;肌肉功能
%V 28
%] 2022292663
%N 4
%J 中医药导报
%Q Effect of Modified Sijunzi Decoction(四君子汤)Combined with Elastic Band Training on Elderly Sarcopenia with Spleen and Stomach Deficiency Syndrome
%X 目的:观察四君子汤加味联合弹力带训练治疗老年肌少症脾胃气虚证的疗效。方法:将100例老年肌少症脾胃气虚证患者随机分为对照组和治疗组,每组50例。对照组患者予老年肌少症的常规干预,治疗组患者在对照组基础上予四君子汤加味联合弹力带训练,均干预8周。比较治疗前后两组患者握力、四肢骨骼肌量指数(ASMI)、简易躯体活动能力评估表(SPPB)评分、营养指标[血清白蛋白(ALB)、前白蛋白(PA)和血清总蛋白(TP)]、中医证候评分,并评定疗效。结果:对照组脱落1例,49例患者完成研究,治疗组50例均完成研究;治疗组总有效率为92.00%(46/50),对照组总有效率为75.51%(37/49),治疗组疗效优于对照组,差异有统计学意义(P<0.05);干预后,两组患者握力、ASMI指数、SPPB评分及营养指标(ALB、PA和TP)均提高(P<0.05),且治疗组患者干预后握力、ASMI指数、SPPB评分及营养指标(ALB、PA和TP)均明显高于对照组,差异均有统计学意义(P<0.05);干预后,两组患者中医证候评分均降低(P<0.05),且治疗组患者干预后中医证候评分明显低于对照组(P<0.05)。结论:四君子汤加味联合弹力带训练治疗老年肌少症脾胃气虚证疗效明显,可有效提高患者的肌肉质量、肌肉功能,改善血清营养指标。
%A 王琪;白晋锋;刘自双;刘芳芳;李国庆;魏莉
%+ 首都医科大学附属北京康复医院,北京 100144
%& 39-44

%0 Journal Article
%@ 1671-5403
%T 中频电刺激联合下肢功率自行车对肌少症患者的治疗效果
%D 2022
%7 20230120
%K 肌少症;中频电刺激;下肢功率自行车
%V 21
%] 2022250204
%N 3
%J 中华老年多器官疾病杂志
%Q Therapeutic effect of medium-frequency electrical stimulation and leg cycle ergometer training for sarcopenia patients
%X 目的:探讨中频电刺激联合下肢功率自行车治疗方案对肌少症患者的治疗效果。方法:纳入2017年9月至2020年2月宜宾市第二人民医院老年医学科住院期间诊断为肌少症94例患者的临床资料。按照治疗方式不同,分为2组。研究组患者46例,给予中频电刺激联合下肢功率自行车治疗;对照组患者48例,不做其他特殊运动处理。4周后评估2组患者治疗前后握力、Berg平衡量表(BBS)评分、移动能力指数(MRMI)评分、简易体能状况量表(SPPB)评分及老年失能评分变化,检测中性粒细胞百分比、C反应蛋白(CRP)、超敏C反应蛋白(hs-CRP)变化。采用SPSS 23.0软件进行数据分析。根据数据类型,组间比较分别采用t检验及χ^(2)检验。结果:治疗4周后,观察组握力与治疗前比较差异无统计学意义[(12.72±1.59)和(10.03±1.38)kg,P>0.05];BBS评分、SPPB评分与治疗前比较,差异有统计学意义[(24.76±4.16)和(17.38±2.90)分、(4.13±0.72)和(2.15±0.48)分,P<0.05];MRMI评分、老年失能评分与治疗前及对照组比较,差异均有统计学意义[(27.57±2.74)和(20.37±2.10)和(20.58±1.47)分、(117.62±12.57)和(93.39±8.74)和(102.26±13.67)分,P<0.05]。治疗4周后,研究组患者中性粒细胞百分比变化与治疗前比较差异无统计学意义[(60.29±3.55)%和(64.30±4.03)%,P>0.05];CRP与治疗前比较差异有统计学意义[(13.56±0.90)vs(16.29±2.07)mg/L,P<0.05];hs-CRP与治疗前及对照组比较,差异均有统计学差异[(4.06±0.94)和(5.31±0.87)和(5.42±0.73)mg/L,P<0.05]。结论:中频电刺激联合下肢功率自行车可以改善肌少症患者的移动能力、平衡功能、体能状况以及肌少症患者体内的炎症状态,使肌少症患者临床获益。
%A 赵旻超;高艳玲;余报;曹桢;段文蓉;金沿欣;何艳
%+ (1)宜宾市第二人民医院康复科,四川宜宾 644000; (2)宜宾市第二人民医院老年医学科,四川宜宾 644000
%& 194-197

%0 Journal Article
%@ 1007-5798
%T 电针联合康复运动训练治疗老年肌少症的临床观察
%D 2022
%7 20220921
%K 老年肌少症;痿证;电针;康复运动训练
%V 30
%] 2022224654
%N 6
%J 中国民间疗法
%Q Clinical observation of electroacupuncture combined with rehabilitation exercise training in treating senile sarcopenia
%X 目的:探讨电针联合康复运动训练治疗老年肌少症的临床疗效。方法:将42例老年肌少症患者随机分为电针组和对照组,每组21例。对照组采用康复运动训练治疗,电针组在对照组基础上联合电针治疗。治疗4周后,比较两组患者治疗前后简易体能状况量表(SPPB)评分、简易5项评分问卷(SARC-F)评分、6min步行距离(6MWT)、握力及骨骼肌指数(RASMI)。结果:治疗后,两组患者SPPB评分均高于治疗前(P<0.05),SARC-F评分低于治疗前(P<0.05),电针组6MWT大于治疗前(P<0.05),且电针组SPPB评分高于对照组(P<0.05),SARC-F评分低于对照组(P<0.05),6MWT大于对照组(P<0.05);两组患者握力及RASMI大于治疗前(P<0.05),且电针组大于对照组(P<0.05)。结论:电针联合康复运动训练可有效改善老年肌少症患者的肌肉质量、肌肉力量,提高其行动能力。
%A 凌绵聪;樊伟;黄文灵;王天磊;潘嘉欣;莫燕丽;刘建浩
%+ 海南省三亚市中医院,海南三亚 572000
%& 52-55

%0 Journal Article
%@ 0254-9026
%T 肌肉衰减综合征与老年人超声最大膈肌活动度的相关性研究
%D 2022
%7 20220921
%K 肌少症;膈肌活动度;超声检查
%V 41
%] 2022207654
%N 2
%J 中华老年医学杂志
%Q The relationship between sarcopenia and the maximum diaphragmatic excursion on ultrasound in the elderly
%X 目的:探讨肌肉衰减综合征与老年人最大膈肌活动度(Dmax)的相关性。方法:从广东省人民医院就诊患者家属中招募老年志愿者(≥60岁),采用超声测量其用力吸气时的Dmax,以四肢肌肉质量指数(ASMI)、上肢握力及日常步行速度作为诊断标准筛选肌肉衰减综合征患者,比较合并与不合并肌肉衰减综合征者在人体学特征,肺通气功能、运动能力及Dmax的差异,并采用线性回归评价肌肉衰减综合征与老年人Dmax的相关性。结果:共纳入145例老年志愿者,年龄(69.47±5.15)岁,其中合并肌肉衰减综合征者28例(19.31%)。肌肉衰减综合征者的体重、ASMI,最大吸气压(Pinmax),最大运动能力(Wmax)及Dmax均低于非肌肉衰减综合征者,差异有统计学意义(均P<0.05)。老年人Dmax与性别、身高、ASMI、握力、步行速度、Pinmax、Wmax的相关性具有统计学意义(r=0.181,0.130,0.322,0.373,0.401,0.134,0.388,P=0.012,0.037,0.009,0.002,0.022,0.009,0.002),在校正性别、年龄。身高及用力肺活量(FVC)后,肌肉衰减综合征与老年人的Dmax仍呈负相关(β=-0.310,P=0.021)。结论:Dmax与老年人Pinmax,Wmax等指标相关,而合并肌肉衰减综合征将增加老年人Dmax下降的风险。
%A 曾斌;何绍冲;梁桂英;刘亚康;王龙平;张鸣生
%+ 广东省医学科学院、广东省人民医院康复医学科/广东省老年医学研究所,广州 510300
%& 196-200

%0 Journal Article
%@ 1673-4246
%T 八段锦联合弹力带训练对老年肌少症康复效果研究
%D 2022
%7 20220921
%K 肌肉衰减征;八段锦;弹力带训练;老年人;康复
%V 44
%] 2022206141
%N 2
%J 国际中医中药杂志
%Q Rehabilitation effect of Baduanjin exercises combined with stretch training with band for elderly patients with sarcopen
%X 目的:观察八段锦联合弹力带训练对老年肌少症患者的康复效果。方法:将符合入选标准的2019年1月-2021年1月本院老年肌少症患者120例按随机数字表法分为2组,每组60例。对照组接受肌少症常规治疗,观察组在对照组基础上给予八段锦联合弹力带训练。2组均干预12周。采用四肢骨骼肌质量指数(Skeletal Muscle Index,SMI)、肌肉握力、简易躯体能力测试表(Short Physical Performance Battery,SPPB)、改良Barthel指数(Modified Barthel Index,MBI)依次对比分析干肌肉活动功能及日常生活能力。结果:观察组干预后SMI指数[(6.77±1.03)kg/m^(2)比(6.35±1.12)kg/m2,t=2.14]、肌肉握力[(23.06±3.48)kg比(19.41±3.79)kg,t=5.50]、SPPB评分[(9.12±2.24)分比(7.85±2.13)分,t=3.18]及MBI评分[(82.43±20.75)分比(64.36±19.42)分,t=4.93]均高于对照组(P<0.05);肌肉活动功能等级的患者分布情况优于对照组干预后(Z=-2.28,P=0.023)。结论:八段锦联合弹力带训练有助于提高老年肌少症患者的肌肉功能和日常活动质量。
%A 李国庆;白晋锋;刘芳芳;燕春花;王琪;魏莉
%+ 首都医科大学附属北京康复医院老年康复中心,北京 100144
%& 164-168

%0 Journal Article
%@ 1004-2814
%T 补中益气汤联合八段锦辅以强化营养支持治疗老年肌少症效果观察
%D 2022
%7 20220921
%K 老年肌少症;补中益气汤;八段锦;强化营养支持
%V 38
%] 2022187683
%N 2
%J 实用中医药杂志
%X 目的:观察补中益气汤联合八段锦辅以强化营养支持治疗老年肌少症的效果。方法:60例以随机数字表法分为研究组与对照组各30例,两组均接受强化营养支持,研究组加用补中益气汤联合八段锦。结果:研究组总有效率高于对照组(P<0.05)。两组握力、肌肉功能、起立-行走计时测试(TGUG)指标均改善,研究组改善幅度大于对照组(P<0.05)。研究组骨折、跌倒等发生率低于对照组(P<0.05)。结论:补中益气汤联合八段锦辅以强化营养支持治疗老年肌少症能够增加肌肉质量,提高肌力水平,预防老年人跌倒、骨折等。
%A 陈欣
%+ 河南中医药大学第一附属医院,河南郑州 450004
%& 179-180

%0 Journal Article
%@ 1007-659X
%T 2型糖尿病合并肌少症发病机制及中医药治疗进展
%D 2022
%7 20220921
%K 2型糖尿病;消渴;肌少症;胰岛素抵抗;炎症反应;脂质沉积;脾肾不足;五脏衰败;八珍汤;补中益气汤
%V 46
%] 2022143438
%N 1
%J 山东中医药大学学报
%Q Pathogenesis of Type 2 Diabetes Mellitus Complicated with Sarcopenia and Progress of Treatment in Traditional Chinese Medicine
%X 2型糖尿病与肌少症关系密切,2型糖尿病可通过多种途径影响肌少症的发生、发展,概述了胰岛素抵抗、炎症反应、脂质沉积、线粒体损伤等与2型糖尿病合并肌少症的相关性。中医认为2型糖尿病归属消渴范畴,病机与五脏衰弱、脾肾不足相关。肌少症在中医理论中可归类于痿证、虚劳的范畴,病机与脾肾不足、五脏衰败有关。两者病本在脾胃亏虚、肾气乏力,治以调理脾肾法,兼以解郁化瘀,临床常用八珍汤、补中益气汤、济生肾气丸、血府逐瘀汤等方剂。并综述了单味中药如黄芪、人参、当归、五味子、枸杞子等能通过抑制炎症因子、改善胰岛素抵抗等途径治疗2型糖尿病合并肌少症。参考文献84篇。
%A 王琪;雷涛;徐媛颖;张翠平;沙雯君;陈琳
%+ (1)上海中医药大学,上海 201203; (2)上海中医药大学附属普陀医院,上海 200062
%& 129-136

%0 Journal Article
%@ 1674-6805
%T 八段锦运动对血液透析并发肌少症患者的干预效果分析
%D 2021
%7 20220921
%K 八段锦;血液透析;肌少症;体力活动;疲乏
%V 19
%] 2022121009
%N 36
%J 中外医学研究
%Q Analysis of Intervention Effect of Baduanjin Exercise on Hemodialysis Patients Complicated with Sarcopenia
%X 目的:分析血液透析前行八段锦运动对血液透析并发肌少症患者体力活动水平及疲乏情况的影响。方法:选取2019年7-12月在广州中医药大学第一附属医院血液净化中心行血液透析治疗并发肌少症的门诊患者115例,每周一、三、五透析患者作为干预组(58例),每周二、四、六透析患者作为对照组(57例)。对照组给予常规护理及运动指导和监督;干预组在对照组的基础上,由血液净化专科护士指导进行透前八段锦运动。干预12周后,比较两组骨骼肌质量指数(SMI)、手握力、日常步行速度、体力活动水平、疲乏等情况。结果:干预后,两组SMI比较,差异无统计学意义(P>0.05);干预后,干预组手握力、日常步行速度、体力活动水平均优于对照组,修改版Piper疲乏量表(RPFS)评分低于对照组,差异均有统计学意义(P<0.05)。结论:在常规护理及运动指导监督基础上,透析前行八段锦运动可以改善患者体能,提高体力活动水平,降低疲乏感。
%A 吴茜;刘泽萍;郭德久
%+ 广州中医药大学第一附属医院,广东广州 510405
%& 97-101

%0 Journal Article
%@ 2095-4352
%T 参苓白术散治疗肌少症患者的临床疗效观察
%D 2021
%7 20220523
%K 参苓白术散;肌少症;腺苷酸活化蛋白激酶;沉默信息调节因子1
%V 33
%] 2021523013
%N 8
%J 中华危重病急救医学
%Q Clinical effect of Senling Baizhu san on patients with sarcopenia
%X 目的:观察参苓白术散治疗脾胃虚弱型肌少症的临床疗效。方法:选择2018年1月至2020年3月杭州市第三人民医院老年科确诊为脾胃虚弱型肌少症的80例住院患者,按照随机数字表法将患者分为对照组和观察组,每组40例。所有患者均采用西医常规治疗,观察组在西医常规治疗基础上加用参苓白术散100mL、每日2次,两组疗程均为12周。记录患者治疗前后握力、步行速度,计算四肢骨骼肌指数(ASMI);采用酶联免疫吸附试验(ELISA)检测血清沉默信息调节因子1(SIRT1)、生长分化因子-8(GDF-8)及胰岛素样生长因子-1(IGF-1)水平;采用实时荧光定量聚合酶链反应(RT-qPCR)检测血清腺苷酸活化蛋白激酶α(AMPK-α)mRNA表达。结果:与治疗前比较,两组患者治疗后握力明显增强,ASMI明显增高,血清IGF-1、SIRT1水平及AMPK-αmRNA表达均明显升高,血清GDF-8水平显著下降,且观察组治疗后上述指标的变化均较对照组更加显著[握力(kg):20.00(15.50,21.00)比18.20(14.93,19.50),ASMI(kg/m^(2)):5.80(5.25,6.00)比5.30(5.20,5.50),IGF-1(μg/L):246.00(229.00,259.50)比207.00(187.00,233.00),SIRT1(ng/L):649.2±38.3比624.6±38.6,AMPK-αmRNA(2^(-ΔΔCt)):0.30±0.03比0.27±0.03,GDF-8(μg/L):13.50(12.00,17.80)比15.60(14.08,19.98),均P<0.05]。而两组治疗前后步行速度差异均无统计学意义[对照组治疗前后为0.56(0.53,0.62)m/s、0.58(0.55,0.62)m/s,观察组治疗前后为:0.58(0.54,0.64)m/s、0.60(0.56,0.65)m/s,均P>0.05]。Spearman相关性分析显示,IGF-1与SIRT1呈正相关(r=0.341,P=0.002),与步行速度呈正相关(r=0.250,P=0.026);ASMI与握力呈正相关(r=0.367,P=0.001)。结论:在西医常规治疗基础上加用参苓白术散治疗脾胃虚弱型肌少症患者效果显著,可为肌少症的治疗提供新的中西医结合思路。
%A 王琴;张彬;林萍;任谦;高康璐;孔程程
%+ 杭州市第三人民医院老年科,浙江杭州 310009
%& 994-998

%0 Journal Article
%@ 1005-0957
%T 热敏灸治疗新型冠状病毒肺炎后肌少症的疗效观察
%D 2021
%7 20220523
%K 灸法;热敏灸;新型冠状病毒肺炎;肌少症;免疫;Berg平衡量表
%V 40
%] 2021493754
%N 9
%J 上海针灸杂志
%Q Efficacy Observation of Heat-sensitive Moxibustion for Sarcopenia After COVID-19
%X 目的:观察热敏灸治疗新型冠状病毒肺炎(COVID-19)后肌少症的临床疗效以及对患者细胞和体液免疫的影响。方法:将100例COVID-19后肌少症患者随机分为对照组和观察组,每组50例。对照组予以营养指导和康复锻炼,观察组在此基础上加用热敏灸治疗。比较两组干预前后相对四肢骨骼肌质量指数(RASMI)、握力、6m行走步速、Berg平衡量表(BBS)评分以及体液免疫指标(IgA、IgM、IgG)和细胞免疫指标(CD3^(+)、CD4^(+),CD4^(+)/CD8^(+))的变化,并比较两组临床疗效。结果:观察组总有效率为92.0%,明显高于对照组的66.0%(P<0.05)。两组干预后RASMI、握力、6m行走步速、BBS评分均较干预前提高,观察者优于对照组,差异有统计学意义(P<0.01)。观察组干预后IgA、IgM、IgG、CD3^(+)、CD4^(+)、CD4^(+)/CD8^(+)水平明显高于干预前及对照组,差异有统计学意义(P<0.01);对照组干预前后上述免疫功能指标比较,差异无统计学意义(P>0.05)。结论:在配合营养指导和康复锻炼的基础上,热敏灸治疗能显著改善COVID-19后肌少症患者中医证候,提高肌肉的质量、力量、功能以及平衡功能,增进患者的免疫机能。
%A 周晶玲;刘飞;陈国超;黄小慧;张爱军
%+ 武汉市武昌医院,武汉 430063
%& 1059-1063

%0 Journal Article
%@ 1008-1070
%T 肠内营养干预对肌少症患者骨代谢及生活质量改善的应用研究
%D 2021
%7 20220523
%K 肠内营养;整蛋白型肠内营养剂;肌少症;骨代谢;生活质量
%V 56
%] 2021473475
%N 9
%J 中国医刊
%Q Application of enteral nutrition intervention on bone metabolism and quality of life improvement in sarcopenia patients
%X 目的:探讨肠内营养干预对肌少症患者骨代谢及生活质量改善的应用价值。方法:选取2019年6月至2020年10月杭州市第三人民医院老年科收治的肌少症患者60例,在日常饮食基础上每天额外增服整蛋白型肠内营养剂进行肠内营养干预。比较分析患者干预前后的体重、四肢骨骼肌质量指数(appendicular skeletal muscle mass index,ASMI)、步速、握力、日常生活活动能力量表(activity of daily living,ADL)评分、疼痛数字评价量表(numerical rating scale,NRS)评分、骨密度T值、维生素D和血钙水平。结果:与干预前比较,肠内营养干预2个月后患者的体重、步速、握力、骨密度T值均显著升高(P<0.05),NRS评分显著下降(P<0.05);肠内营养干预3个月后患者的体重、ASMI、步速、握力、ADL评分、骨密度T值、维生素D和血钙水平均显著升高(P<0.05),NRS评分显著下降(P<0.05)。与肠内营养干预2个月时比较,肠内营养干预3个月后患者的体重、ASMI、ADL评分、骨密度T值、维生素D和血钙水平均显著升高(P<0.05),NRS评分显著下降(P<0.05)。结论:肠内营养干预可以改善肌少症患者的骨代谢,增加患者的骨骼密度,缓解疼痛症状,提高运动能力,显著改善患者的生活质量。
%A 高康璐;马璐瑶;王琴;林萍
%+ 杭州市第三人民医院老年科,浙江杭州 310009
%& 999-1002

%0 Journal Article
%@ 2095-0616
%T 补中益气汤加减对老年肌少症患者炎症因子的影响
%D 2021
%7 20220523
%K 补中益气汤;老年肌少症;白细胞介素-6;肿瘤坏死因子-α
%V 11
%] 2021473001
%N 16
%J 中国医药科学
%Q Effect of Buzhong Yiqi Decoction on inflammatory factors in elderly patients with sarcopenia
%X 目的:探讨补中益气汤加减对老年肌少症患者炎症因子的影响。方法:本研究选取广东省深圳市中医院2019年7月至2020年11月收治的40例60岁以上肌少症患者,按照随机数字表法方法分为治疗组(n=20)与对照组(n=20)。对照组给予基础干预,治疗组在对照组基础上同时予以补中益气汤加减治疗。比较两组的治疗前后的白细胞计数、丙氨酸氨基转移酶、血肌酐指标变化及白细胞介素-6(IL-6)、肿瘤坏死因子-α(TNF-α)变化。结果:用药2个月后,两组治疗前后白细胞计数、丙氨酸氨基转移酶、血肌酐指标比较,差异均无统计学意义(P>0.05);两组治疗前IL-6、TNF-α比较,差异无统计学意义(P>0.05);用药2个月后,两组患者的IL-6、TNF-α均低于治疗前,且治疗组低于对照组,差异均有统计学意义(P<0.05)。结论:补中益气汤加减治疗老年肌少症安全,无不良反应,并能更有效地降低IL-6、TNF-α的水平。
%A 陈颖颖;温春瑜;焦萁荟
%+ (1)深圳市中医院综合病区,广东深圳 518000; (2)深圳市中医院心血管科,广东深圳 518000
%& 13-16

%0 Journal Article
%@ 1006-0979
%T 曲美他嗪联合八珍汤治疗老年慢性心衰合并肌少症的临床效果
%D 2021
%7 20210910
%K 曲美他嗪;八珍汤;老年慢性心衰;肌少症;临床效果
%V 40
%] 2021293315
%N 3
%J 内蒙古中医药
%X 目的:探究曲美他嗪联合八珍汤治疗老年慢性心衰(CHF)合并肌少症的临床效果。方法:选取2017年1月-2020年2月我院收治的98例老年慢性心衰合并肌少症患者,随机分为参照组和研究组,各49例。参照组予以常规营养支持、曲美他嗪治疗,研究组在此基础上加用八珍汤治疗,观察两组治疗前后心功能、肌肉质量及肌肉力量。结果:治疗前,两组心脏超声、肌肉质量、肌肉力量差异无统计学意义(P>0.05);治疗后,研究组LVEF、肌肉质量、肌肉力量高于参照组,而LVEDD、LVESD低于参照组,差异有统计学意义(P<0.05)。结论:曲美他嗪联合八珍汤治疗CHF合并肌少症效果良好,可以改善心功能、提升肌肉质量及肌肉力量。
%A 郝媛媛;胡石甫
%+ 天津市西青医院,天津 300100
%& 57-58

%0 Journal Article
%@ 1007-3213
%T 从肠肌轴学说与“脾主肌肉”的关系探究针灸治疗压力性尿失禁的可行性
%D 2021
%7 20210910
%K 针灸;压力性尿失禁;肠肌轴学说;肠道菌群;脾主肌肉;健脾益气法
%V 38
%] 2021267818
%N 4
%J 广州中医药大学学报
%Q Explore the Feasibility of Acupuncture-Moxibustion Treatment for Stress Urinary Incontinence Based on Relationship between the Theory of Gut-Muscle Axis and The Spleen Nourishes the Muscles
%X 针灸治疗压力性尿失禁(SUI)疗效可靠,近年来对针灸治疗SUI的治疗机制多有研究,其研究角度多从促神经修复和改善结缔组织胶原代谢进行阐释。肠道菌群与肌肉的关系是近年来的研究热点之一,现已广泛应用于肌少症等疾病研究,用于阐述骨骼肌与肠道菌群之间的关系。盆底肌肉薄弱和功能下降是SUI发病的重要原因,肠道菌群失调或可引起盆底肌肉薄弱和功能下降,肠道菌群或可成为增加盆底肌肉支持力和功能,改善SUI的机制之一。基于肠肌轴理论,通过健脾益气法针灸来改善盆底肌肉功能以防治SUI。
%A 冉津川;李超楠;陈炳力;舒文;阮铄荃;段婷婷;侯文光
%+ 上海中医药大学附属岳阳中西医结合医院,上海 200437
%& 723-729

%0 Journal Article
%@ 1003-5028
%T 基于“治痿独取阳明”探讨电针疗法在老年肌少症中的应用
%D 2020
%7 20210603
%K “治痿独取阳明”;老年肌少症;电针疗法;足三里穴;环跳穴;承山穴
%V 40
%] 2020731763
%N 11
%J 河南中医
%Q Discussion About the Application of Electroacupuncture Therapy in Senile Sarcopenia Based on "Treating Flaccidity Only Taking Yangming Meridian"
%X 早期痿证,病情多轻浅,治疗上多选用手足阳明经穴进行治疗。痿证日久,伤及脏腑,病势缠绵,故治疗上以"主取阳明"为法,在调护阳明经脉气血的同时,兼顾他经他脏。对于病情复杂,病变范围广、疾病所涉经脉较多的痿证,不仅治疗上重点选择阳明经,并酌情选用手足三阴经、手足太阳经、手足少阳经施治,此谓之"多经多穴"法。在选阳明经穴的基础上,同时选用督脉以及八会穴、原穴等有特殊作用的穴位。电针治疗老年肌少症的作用机制有:改善低炎症状态,调节激素水平,影响细胞自噬与凋亡,调节泛素-蛋白酶系统。老年肌少症病因病机复杂,病机关键在于肌肉瘦消无力,筋脉痿弱不用,故取穴总不离阳明经。电针治疗老年肌少症,阳明经足三里穴是治疗中的基础穴位,也是必选穴位,在此基础上加用足少阳经环跳穴、足太阳经承山穴等。
%A 刘传凤;吴雨潇;王欣欣;余泽芸;贾华楠;钟文
%+ (1)成都中医药大学附属医院,四川成都 610075; (2)成都中医药大学,四川成都 611137
%& 1725-1728

%0 Journal Article
%@ 1672-951X
%T 中医药治疗老年肌少症研究进展
%D 2020
%7 20210603
%K 中医药;特色疗法;肌少症;老年;综述
%V 26
%] 2020674566
%N 13
%J 中医药导报
%Q A Review on the Treatment of Chinese Medicine for Elderly Patients with Sarcopenia
%X 总结了中医药治疗老年肌少症的相关研究,包括中药复方和针刺、推拿、太极拳、易筋经等传统特色疗法治疗老年肌少症的中医药理论依据和临床疗效。认为中医药治疗可以缓解肌少症的症状,增强肌力、肌量,改善步态、步速与日常生活能力,提高行走能力与平衡能力。诸多研究表明中医药治疗老年肌少症疗效确切,副作用少,为临床治疗方案提供了新思路。
%A 齐涵;赵洪欣;孟凯华;张泽
%+ (1)辽宁中医药大学,辽宁沈阳 110847; (2)辽宁中医药大学附属医院,辽宁沈阳 110032
%& 179-182

%0 Journal Article
%@ 2096-1278
%T 痿三针联合康复训练改善肌少症下肢运动功能和平衡及步行能力
%D 2020
%7 20210603
%K 平衡能力;步行能力;肌少症;痿三针
%V 28
%] 2020670635
%N 10
%J 临床研究
%X 目的:观察"痿三针"疗法对肌少症患者下肢运动功能和平衡及步行能力的疗效。方法:符合诊断标的60例患者来源于郑州大学附属郑州中心医院(包括门诊6例及住院43例)、新郑市公立人民医院(包括门诊1例及住院10例)2017年9月-2019年5月的就诊患者,随机分成治疗组和对照组,每组30例,治疗组用"痿三针"治疗,对照组用常规针刺治疗,治疗前、后分别评估患者Fugl-Meyer下肢运动功能评分、Berg平衡量表、步速。结果:两组患者与治疗前相比,Fugl-Meyer下肢运动功能评分、Berg平衡量表、步速均提高,差异显著(P<0.05)。且治疗组改善情况优于对照组(P<0.05)。结论:痿三针疗法对肌少症患者下肢运动功能和平衡及步行能力的改善优于普通针刺组。
%A 刘碧原;王景信;张艳;毛璐熙;赵莎莎;范慧轩;甄佳美;郭艳丽
%+ (1)郑州大学附属郑州中心医院康复医学科,河南郑州 450000; (2)新郑市公立人民医院康复医学科,河南郑州 450000
%& 136-137

%0 Journal Article
%@ 1674-8999
%T 含有人参的中药复方在老年综合征中的应用举隅
%D 2020
%7 20210311
%K 老年综合征;人参;中药复方
%V 35
%] 2020585790
%N 8
%J 中医学报
%Q Analysis on Application of Herbal Compounds Containing Renshen(Ginseng)in Treating Geriatric Syndromes
%X 人参为补虚之要药,主治病证繁多,且多以气虚病证为主。将人参应用到老年综合征各系统疾病的治疗中,不仅能够缓解症状,更可调节老年患者气虚、阳虚之体质,补益正气,从整体改善患者身体状况。临床常用黄龙汤、六君子汤、补中益气汤等治疗便秘、泄泻、肌少症等脾胃病;麦门冬汤等治疗咳嗽、肺痿等肺系疾病;炙甘草汤、天王补心丹等治疗心悸、失眠、痴呆等心系疾病;春泽汤等治疗小便失常等肾系疾病;益气聪明汤等治疗耳鸣、视物不清等肝系疾病。
%A 蔡芮桐;陈民;齐涵;张泽
%+ (1)辽宁中医药大学,辽宁沈阳 110847; (2)辽宁中医药大学附属医院,辽宁沈阳 110032
%& 1639-1642

%0 Journal Article
%@ 1673-7555
%T 补中益气汤治疗老年肌少症的临床疗效及对C反应蛋白的影响
%D 2020
%7 20210311
%K 补中益气汤;老年肌少症;C反应蛋白
%V 15
%] 2020524120
%N 14
%J 中国实用医药
%X 目的:运用补中益气汤加减治疗老年肌少症患者,观察临床疗效及对C反应蛋白的影响。方法:60例老年肌少症患者,随机分为对照组与治疗组,各30例。对照组患者采用基础干预,治疗组在采用基础干预的同时服用补中益气汤加减治疗。比较两组患者的中医症候疗效,治疗前后的肌肉质量、肌肉力量、肌肉功能、C反应蛋白。结果:治疗组中医症候总有效率93.33%高于对照组的73.33%,差异具有统计学意义(P<0.05)。治疗前,两组患者的肌肉质量、肌肉力量、肌肉功能比较,差异均无统计学意义(P>0.05);治疗后,两组患者的肌肉质量、肌肉力量、肌肉功能均较本组治疗前升高,且治疗组升高程度优于对照组,差异均具有统计学意义(P<0.05)。治疗前,两组患者的C反应蛋白水平比较,差异无统计学意义(P>0.05);治疗后,两组患者的C反应蛋白水平均较本组治疗前降低,且治疗组(3.12±2.21)mg/L低于对照组的(4.05±1.01)mg/L,差异均具有统计学意义(P<0.05)。结论:补中益气汤加减联合基础干预治疗老年肌少症优于单纯基础干预治疗,能有效改善老年肌少症患者的临床症状,包括中医症候疗效的改善和肌肉质量、肌肉力量、肌肉功能的改善,安全有效,无不良反应。采用补中益气汤联合治疗的患者C反应蛋白降低更明显。
%A 陈颖颖
%+ 深圳市中医院心血管内科, 518000
%& 166-168

%0 Journal Article
%@ 1003-8914
%T 痿三针联合康复训练治疗肌少症临床观察
%D 2020
%7 20200807
%K 表面肌电;肌少症;痿三针
%] 2020415884
%N 1
%J 光明中医
%Q Analysis on the Therapeutic Effect of Wei Three-needle Combined with Rehabilitation Training on Myopathy
%X 目的:观察"痿三针"疗法对肌少症患者步行能力、肌力、平衡功能的疗效。方法:将符合诊断标准的60例患者随机分成治疗组和对照组,每组30例,治疗组用"痿三针"治疗,对照组用常规针刺治疗,10d后分别测试2组患者步行速度、站位平均动摇速度、双下肢腓肠肌表面肌电数值。结果:与治疗前相比,治疗后2组患者步行速度升高,站位平均重心动摇速度下降,双下肢腓肠肌表面肌电数值提高(P<0.05),且治疗组改善优于对照组(P<0.05)。结论:痿三针疗法对肌少症的疗效优于常规针刺。
%A 刘碧原;王景信;毛璐熙;张艳;范慧轩;甄佳美
%+ 郑州大学附属郑州中心医院康复治疗科,河南郑州 450000
%& 70-72

%0 Journal Article
%@ 1674-8972
%T 神经肌肉电刺激治疗肌少症所致吞咽障碍的疗效评估
%D 2020
%7 20200804
%K 神经肌肉;电刺激;肌少性吞咽障碍;临床疗效
%V 29
%] 2020330743
%N 1
%J 癫痫与神经电生理学杂志
%Q Effect of neuromuscular electrical stimulation on sarcopenic dysphagia
%X 目的:探究神经肌肉电刺激治疗肌少症所致吞咽功能障碍患者的临床疗效。方法:将2017年3月至2018年9月期间在重庆市第九人民医院接受诊治的80例肌少症所致吞咽障碍患者作为研究对象,将其分为两组,干预组患者给予神经肌肉电刺激治疗,对照组患者给予健康教育,基础治疗用药相同,分别在治疗前后对患者的吞咽功能情况进行评估,并统计患者的治疗效果。结果:治疗后两组患者标准吞咽功能评价量表评分均有所下降,但干预组患者下降幅度更为明显,经比较差异有统计学意义(P<0.05);干预组患者治愈率(32%)和总有效率(95%)显著优于对照组患者治愈率(15%)和总有效率(70%),经比较差异有统计学意义(P<0.05)。结论:将神经肌肉电刺激应用于肌少症所致吞咽障碍的临床治疗当中,可有效改善患者的吞咽功能,效果显著。
%A 何国金;杨馨;金茜;黄敏;何茂锐;陈强
%+ 重庆市第九人民医院全科医疗科,重庆 400700
%& 4-7

%0 Journal Article
%@ 1001-2001
%T 浙江省社区康复服务资源的现状调查
%D 2019
%7 20191110
%K 社区康复;可持续发展;浙江省
%V 34
%] 2019365940
%N 5
%J 中国康复
%Q Research report on the current situation of community rehabilitation in Zhejiang province
%X 目的:调查浙江省社区康复现状,为我省社区康复的可持续发展战略提供依据。方法:选择全省11个地区64家社区卫生服务中心及农村乡镇卫生服务中心发放问卷,对社区康复服务部门人力资源状况、开展的主要康复治疗项目、康复站规模及设施等方面进行调查。结果:64家社区共回收有效问卷64份,从调查结果我们可以发现研究生、本科生、大专、中专生分别占5.3%、23.0%、41.1%、30.6%。康复工作人员中康复医师、康复治疗师、全科医师、康复护士分别占25.9%、38.5%、10.5%、27.1%。职称以高级、中级、初级分别占5.3%、34.3%、38.7%。以康复为全职的占36.2%,曾参加康复专科继续教育学习占43.2%。康复治疗(训练)方面主要以上下肢运动能力训练、推拿按摩为主;针对≥65周岁老年人生活及运动方式指导,包括预防跌倒训练、提高老年人心肺(有氧)能力的训练、改善老年肌少症的训练等3个项目所占比例均偏低,分别为20.3%、18.7%、23.4%。社区卫生机构拥有较多的康复硬件是电针仪、远红外治疗仪、轮椅、颈腰牵引床,分别为81.2%、76.5%、73.4%、60.9%。踝关节矫形器、木丁板、PT凳、矫姿镜拥有率最低,分别是29.6%、29.6%、31.2%、32.8%。结论:社区康复在我省得到健康的发展,体系也逐渐完善,但目前仍面临着许多挑战。建议今后我省社区康复服务能力建设过程中将转变社区康复从业人员的服务理念及社区居民的康复意识作为努力方向,重点提高社区康复服务体系中针对老年人及慢病患者的相应服务能力;此外,政策进一步向社区康复倾斜,争取从技术上,资源配备上、人才管理以及中远期规划上保证和提高社区康复应有的质和量,为我省社区康复事业提供一条可持续发展道路。
%A 章睿;吴燕萍;谢淑珍;陈定湾;林坚;沈振鹏;王晓红;蔡利锋;毛文英
%+ (1)浙江中医药大学,杭州 310053; (2)浙江医院,杭州 310030; (3)浙江省卫生计生委基层处,杭州 310006; (4)浙江省杭州市西湖区古荡社区卫生服务中心,杭州 310013; (5)浙江省杭州医学院,杭州 310053; (6)浙江省杭州市灵隐社区卫生服务中心,杭州 310012
%& 274-277

%0 Journal Article
%@ 1671-4040
%T 补中益气汤加减辅助治疗老年肌少症的临床疗效观察
%D 2018
%7 20190520
%K 肌少症;老年;补中益气汤;躯体功能
%V 18
%] 2018517681
%N 7
%J 实用中西医结合临床
%X 目的:观察补中益气汤加减辅助治疗老年肌少症的临床效果。方法:选取2016年1月－2017年12月在我院确诊为老年肌少症患者60例为研究对象,按随机数字表法分为观察组和对照组,各30例。对照组给予常规干预,观察组在对照组基础上给予补中益气汤加减辅助治疗,观察两组患者肌肉质量、肌肉力量、身体活动功能和日常生活能力。结果:治疗前,两组肌肉质量、肌肉力量、身体活动功能和日常生活能力比较均无显著性差异,P>0.05;治疗后,观察组肌肉质量、肌肉力量、身体活动功能和日常生活能力均明显高于对照组,P<0.05。结论::补中益气汤加减辅助治疗老年肌少症,可有效改善患者躯体功能,增强肌肉力量,改善日常生活能力。
%A 温春瑜;陈颖颖;彭鹏;焦萁荟
%+ 广东省深圳市中医院,深圳 518033
%& 72-73

%0 Journal Article
%@ 1001-1242
%T 推拿结合抗阻运动对骨骼肌衰减症患者日常生活活动能力的影响
%D 2016
%7 20170128
%K 推拿;抗阻运动;骨骼肌衰减症;日常活动活动能力
%V 31
%] 2016549180
%N 9
%J 中国康复医学杂志
%Q Effects of Chinese massage and resistance exercise on ADL in elderly Chinese sarcopenic men
%X 目的:探讨推拿或/和抗阻运动对骨骼肌衰减症男性患者骨骼肌质量、肌力、步速及日常活动能力的影响,为推拿及运动防治老年人骨骼肌衰减症提供科学依据。方法:按照亚洲肌少症工作组确定的骨骼肌衰减症诊断标准筛选符合标准的骨骼肌衰减症老年男性志愿者24例,并随机平均分为4组:对照组(C)、推拿组(M)、抗阻运动组(R)、推拿结合抗阻运动组(MR)。M组受试者接受推拿功法易筋经(20min)和推拿手法(20min)干预;R组受试者分别进行60%-80%1RM、3组/次、组间歇3－5min的板凳深蹲(20min)和弹力带练习(20min);MR组受试者交替进行推拿干预和抗阻运动练习。干预频率为3次/周,为期8周,8周前后分别测量受试者四肢骨骼肌质量、肌力、步速及Barthel指数,并进行统计分析。结果:①组内比较发现:C组仅步速显著下降;M组和R组握力、步速和Barthel指数显著提高,四肢骨骼肌质量指数(appendicular skeletal muscle mass,ASMI)值小幅提升但无差异显著性;MR组四项测量指标均显著升高。②组间比较发现:与C组相比,M组仅步速显著提高,R组除ASMI值外均显著提高,MR组四项测量指标均显著升高;与M组相比,MR组四项测量指标均显著升高;与R组相比,MR组仅Barthel指数显著提高。结论:推拿结合抗阻运动能有效提升骨骼肌衰减症患者骨骼肌质量、肌力、步行能力及整体活动能力,且其改善效果优于单纯进行推拿或抗阻运动干预,是值得推荐的治疗骨骼肌衰减症的有效手段之一。
%A 赵永军;张育民;郭艳花;窦艳丽;赵娟;何玉秀
%+ (1)河北师范大学人体运动生物信息省级重点实验室, 石家庄 050024; (2)吕梁学院体育系; (3)太原市和平医院
%& 989-994

%0 Journal Article
%@ 1003-5370
%T 太极拳对老年人下肢肌力及功能的影响
%D 2016
%7 20161207
%K 老年人;太极拳;肌力;平衡能力;肌少症
%V 36
%] 2016227115
%N 1
%J 中国中西医结合杂志
%Q Effect of Tai Ji Quan Training on Strength and Function of Lower Limbs in the Aged
%X 目的:观察太极拳练习对老年人下肢肌力及功能的影响。方法:选择60名老年人按随机数字表分为太极拳练习组(干预组)与健康知识宣教组(对照组),分别予18个月24式太极拳练习(60min/次,5次/周)与老年肌少症相关病因、发病机制及防治措施宣教(120min/次,1次/月)。比较两组髂腰肌、股四头肌、胫前肌、腘绳肌最大等长肌力,5次起坐、计时"起立-行走"(Time Up and Go Test,TUGT)、闭目单腿站立时间、Berg平衡量表评分。结果:与本组干预前比较,干预组双侧髂腰肌、股四头肌、胫前肌力均增高(P<0.05),平均增长量(率)依次为5.5kg(16.9%)、5.5kg(26.2%)、8.5kg(36.2%);TUGT、5次起坐时间分别缩短1.3s(16.7%)、0.9s(14.5%),双侧闭目单腿站立时间增加8.4s(左)、9.1s(右),Berg平衡量表评分增加4.3%,差异均有统计学意义(P<0.05)。与对照组同期比较,干预组干预后双侧股四头肌、胫前肌力增加(P<0.01);TUGT、5次起坐、双侧闭目站立时间、Berg平衡量表评分均改善(P<0.05)。结论:太极拳能改善老年人髂腰肌、股四头肌、胫前肌肌力,提高平衡及活动能力,可能防治老年人肌少症。
%A 朱亚琼;彭楠;周明
%+ (1)中国人民解放军总医院康复医学科,北京 100853; (2)中国人民解放军总医院康复医学中心,北京 100853
%& 49-53

%0 Journal Article
%@ 1673-9701
%T 八珍汤联合基础干预治疗老年肌少症临床疗效观察
%D 2016
%7 20161130
%K 老年肌少症;肌肉质量;肌肉力量;肌肉功能;八珍汤;基础干预
%V 54
%] 2016463044
%N 16
%J 中国现代医生
%Q Clinical effect observation of Bazhen soap combined with basic interven-tion in the treatment of elderly sarcopenia
%X 目的:探讨应用八珍汤联合营养支持、体育锻炼治疗老年肌少症的临床疗效。方法:选择2014年12月－2015年12月在我院就诊的肌少症患者90例,随机分为治疗组和对照组,两组均应用基础干预即营养支持与体育锻炼,治疗组同时采用八珍汤为基础方加减,总疗程为12周,比较两组的疗效及两组患者治疗前后肌量、肌力、肌功能的变化。结果:治疗组总有效率93.33%,高于对照组的75.56%,组间比较,差异有统计学意义(P<0.05)。治疗前,两组患者的肌量、肌肉力量及肌肉功能比较,差异无统计学意义(P>0.05);治疗后,两组患者的肌肉质量、肌肉力量、肌肉功能较治疗前显著提高,且治疗组患者的肌肉质量、肌肉力量和肌肉功能改善显著优于对照组,差异有统计学意义(P<0.05)。结论:八珍汤联合营养支持、体育锻炼治疗老年肌少症疗效确切,可以显著改善患者的肌肉质量、肌肉力量和肌肉功能,提高了临床治疗效果,值得进一步推广和应用。
%A 任璇璇;姚惠;汪涛
%+ 浙江医院中医科,浙江杭州 310017
%& 127-130

  
