# Supplementary material for: The potential of acupuncture in treating sarcopenia: a systematic review and meta-analysis of randomized controlled trials
Source: Front Public Health. 2025 Nov 10;13:1696030. doi: 10.3389/fpubh.2025.1696030 (PMC12640850; doi:10.3389/fpubh.2025.1696030)
Supplement: Supplementary file 1 [file Supplementary_file_1.zip › Supporting Information/1.Data/Supporting Information/PRISMA_2020_flow_diagram.docx]

**Identification of studies via databases and registers**

Duplicate records removed

(n = 988)

Records identified from:

Database searching (n = 2094)

**Identification**

Records sought for retrieval

(n = 1106)

Records excluded:

Unrelated studies (n = 281)

Basic research (n = 384)

Review articles (n = 324)

Case reports (n = 8)

**Screening**

Records assessed for eligibility

(n = 109)

Records excluded:

Unable to extract valid data

(n =99)

Records of included studies

(n = 10)

**Included**
