# Supplementary material for: The potential of acupuncture in treating sarcopenia: a systematic review and meta-analysis of randomized controlled trials
Source: Front Public Health. 2025 Nov 10;13:1696030. doi: 10.3389/fpubh.2025.1696030 (PMC12640850; doi:10.3389/fpubh.2025.1696030)
Supplement: Supplementary file 1 [file Supplementary_file_1.zip › Supporting Information/2.Tables/Table 1. Main characteristics of included studies.docx]

Table 1. Main characteristics of included studies.

| Author/Year | Country | Diagnostic criteria | Sample Size (EG/CG) | Mean Age  (EG/CG, years) | Intervention EG | Intervention CG |
| --- | --- | --- | --- | --- | --- | --- |
| Zhendi Feng 2023 | China | CECDTES 2021 | 130/130 | 56±11/  57±11 | Acupuncture (LI4, SI3, SJ5, LI10, LI11, LI14, SJ13, GB31, ST34, SP10, ST36, GB34, SP9, ST40, BL57, SP6, BL60, KI3, LR3, KI1) combined with conventional treatment | Conventional treatment |
| Gisele Soares 2019 | Brazil | EWGSOP 2010 | 11/4 | 72±7.9/  63.5±3.3 | Acupuncture (LI4, KI3, SP6, GB34, ST36) | No intervention |
| Weibo Gu 2022 | China | AWGS 2019 | 25/27 | 77.68±6.24/  76.81±5.78 | Acupuncture (LI11, LI10, ST31, ST32, ST34, ST36, ST37, ST39, ST41) combined with exercise | Conventional treatment |
| Miancong Ling 2022 | China | AWGS 2019 | 21/21 | 64.7±6.2/  65.3±5.8 | Electroacupuncture (CV6, CV4, ST36, GB34, LU5, LI11, SP6, GV14, SP9, ST44, CV12, ST25, SP3, LR3, KI3) combined with exercise | Exercise |
| Biyuan Liu 2020 | China | IWGS 2011 | 30/30 | 68.12±5.84/  70.17±4.56 | Weisanzhen Acupuncture (ST36, KI3, SP6) combined with exercise | Conventional treatment |
| Sufan Ma 2023 | China | AWGS 2014 | 30/30 | 65±5.19/  68±7.41 | Electroacupuncture (LI14, LI11, LI4, ST31, ST34, ST36, GB34, SP6) combined with nutritional support | Nutritional support |
| Hui Pang 2023 | China | AWGS 2019 | 30/30 | 70.2±4.48/  70±4.75 | Mediating acupuncture (ST36, KI3, SP6) combined with conventional treatment | Conventional treatment |
| Xin Zhou 2018 | China | AWGS 2014 | 23/25 | 70.35±5.36/  68.8±5.08 | Electroacupuncture (LI14, LI11, ST31, ST34) combined with nutritional support | Nutritional support |
| Jing Yang 2022 | China | AWGS 2014 | 32/31 | 56±8/  57±6 | Warming needle moxibustion (LI14, LI11, LI4, ST31, ST34, ST36, GB34, SP6) combined with conventional treatment | Conventional treatment |
| Yanli Zhang 2024 | China | CECDTES 2021 | 47/47 | 72.84±5.81/  71.91±6.03 | Acupuncture (LI4, LI10, LI11, LI14, LI15, ST31, ST32, ST35, ST36, ST37, ST39, ST39, ST40, GB34, CV4, ST25, CV12) combined with conventional treatment | Conventional treatment |

Notes. EG = Experimental group, CG = Control group. CECDTES = Chinese expert consensus on diagnosis and treatment for elderly with sarcopenia 2021, Conventional treatment: wellness education, nutritional support, daily activities or exercise.
