# Supplementary material for: The potential of acupuncture in treating sarcopenia: a systematic review and meta-analysis of randomized controlled trials
Source: Front Public Health. 2025 Nov 10;13:1696030. doi: 10.3389/fpubh.2025.1696030 (PMC12640850; doi:10.3389/fpubh.2025.1696030)
Supplement: Supplementary file 1 [file Supplementary_file_1.zip › Supporting Information/2.Tables/Table 2. Distribution of the RoB 2 graph.docx]

| Table 2. Distribution of the RoB 2 graph | | | | | | |  |  |  |  |  |  |
| --- | --- | --- | --- | --- | --- | --- | --- | --- | --- | --- | --- | --- |
| Study ID | D1 | D2 | D3 | D4 | D5 | Overall |  |  |  |  |  |  |
| Zhendi Feng2023 | 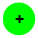 | 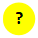 | 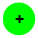 | 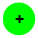 | 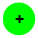 | 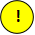 |  |  |  |  |  |  |
| Gisele Soares 2019 | 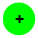 | 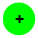 | 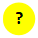 | 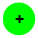 | 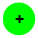 | 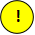 |  |  |  |  |  |  |
| Weibo Gu 2022 | 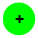 | 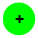 | 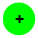 | 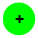 | 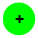 | 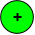 |  |  |  |  |  |  |
| Miancong Ling 2022 | 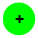 | 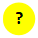 | 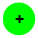 | 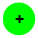 | 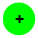 | 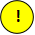 |  |  |  |  |  |  |
| Biyuan Liu 2020 | 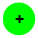 | 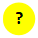 | 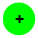 | 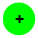 | 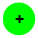 | 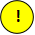 |  |  |  |  |  |  |
| Sufan Ma 2023 | 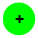 | 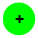 | 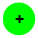 | 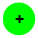 | 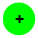 | 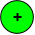 |  |  |  |  |  |  |
| Hui Pang 2023 | 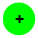 | 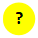 | 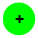 | 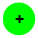 | 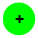 | 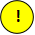 |  |  |  |  |  |  |
| Xin Zhou 2018 | 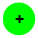 | 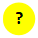 | 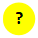 | 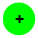 | 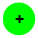 | 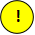 |  |  |  |  |  |  |
| Jing Yang 2022 | 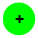 | 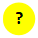 | 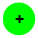 | 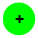 | 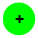 | 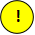 |  |  |  |  |  |  |
| Yanli Zhang 2024 | 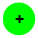 | 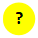 | 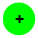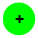 | 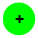 | 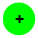 | 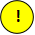 |  |  |  |  |  |  |

| 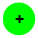 | Low risk |  |
| --- | --- | --- |
| 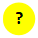 | Some concerns |  |
| 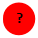 | High risk |  |
|  |  |  |
| D1 | Randomization process |  |
| D2 | Deviations from intended interventions |  |
| D3 | Missing outcome data |  |
| D4 | Measurement of the outcome |  |
| D5 | Selection of the reported result |  |
|  |  |  |
