# Supplementary figures and images for: The potential of acupuncture in treating sarcopenia: a systematic review and meta-analysis of randomized controlled trials
Source: Front Public Health. 2025 Nov 10;13:1696030. doi: 10.3389/fpubh.2025.1696030 (PMC12640850; doi:10.3389/fpubh.2025.1696030)

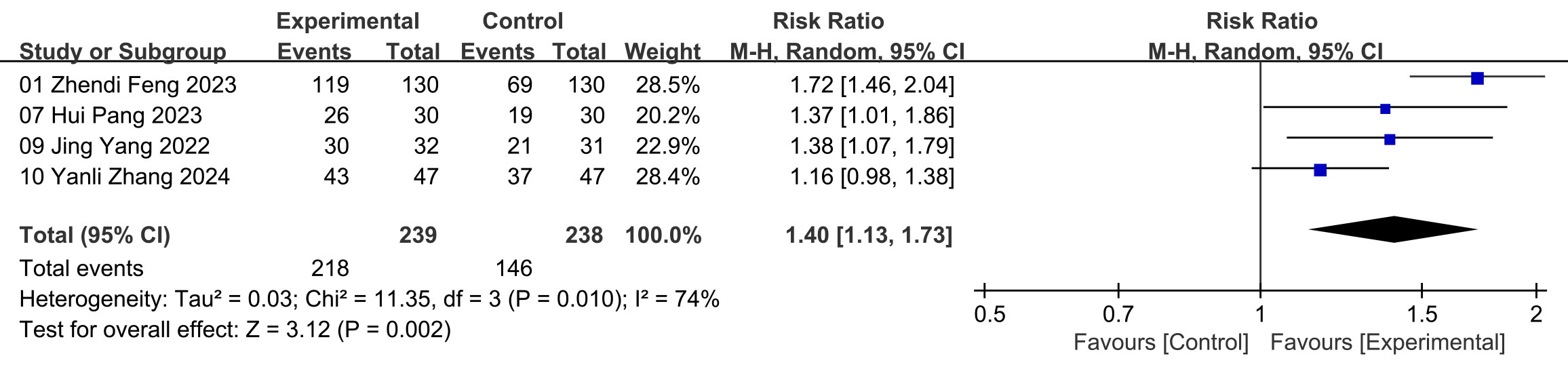

Supplement: Supplementary file 1 [file Supplementary_file_1.zip › Supporting Information/3.Supplementary figure/Figure S1. Forest plot of total efficiency with random-effects model.tif]

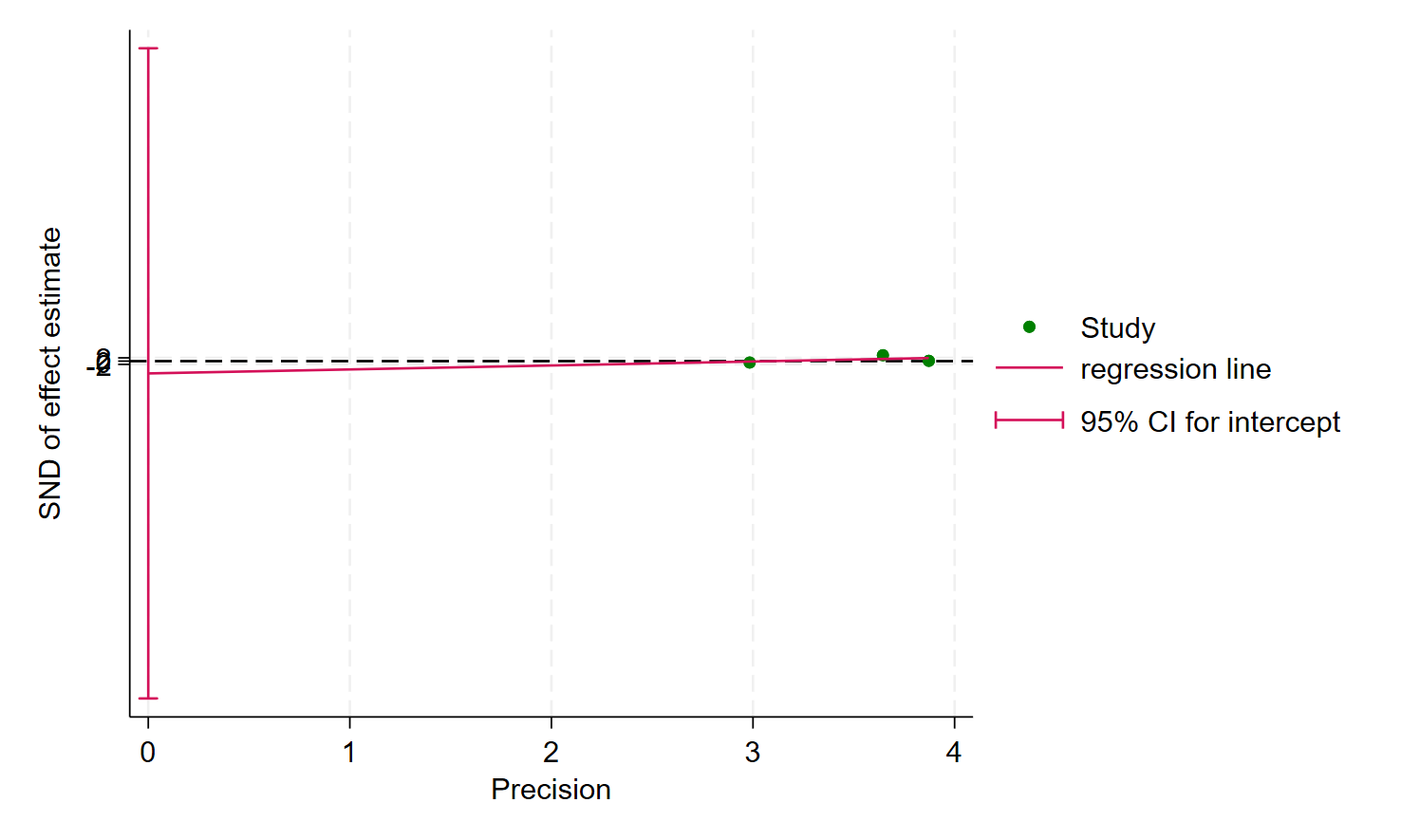

Supplement: Supplementary file 1 [file Supplementary_file_1.zip › Supporting Information/3.Supplementary figure/Figure S10. Egger’s test plot of usual gait speed.tif]

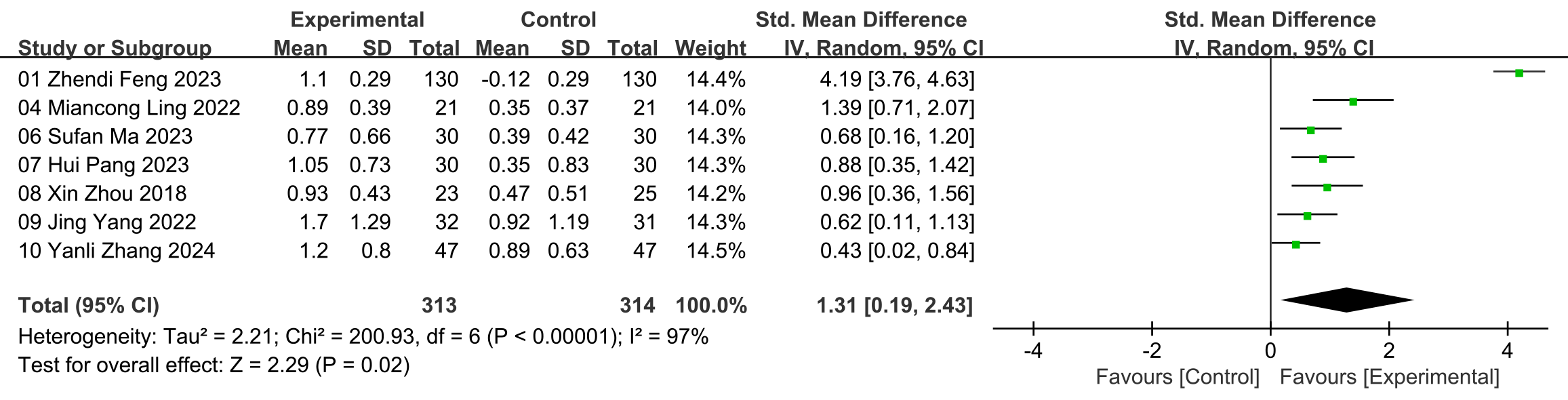

Supplement: Supplementary file 1 [file Supplementary_file_1.zip › Supporting Information/3.Supplementary figure/Figure S2. Forest plot of muscle mass with random-effects model.tif]

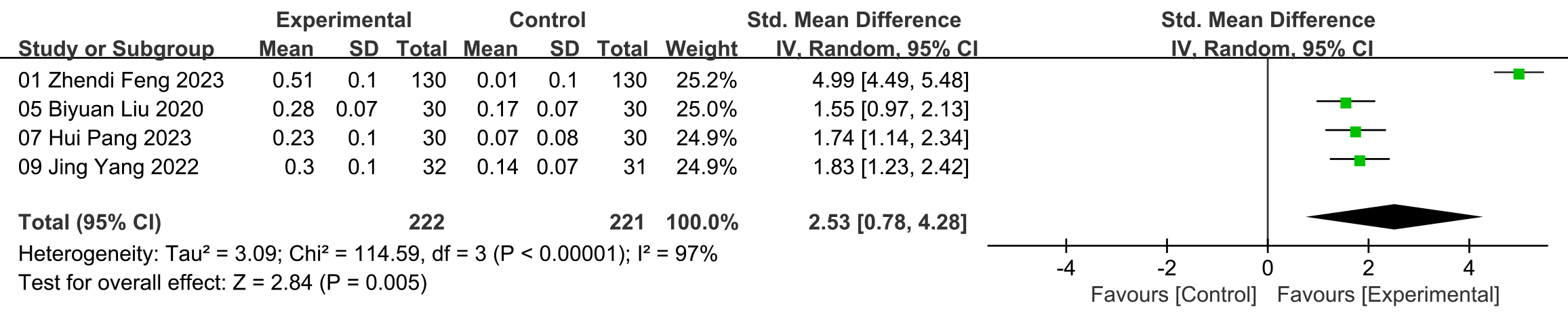

Supplement: Supplementary file 1 [file Supplementary_file_1.zip › Supporting Information/3.Supplementary figure/Figure S3. Forest plot of usual gait speed with random-effects model.tif]

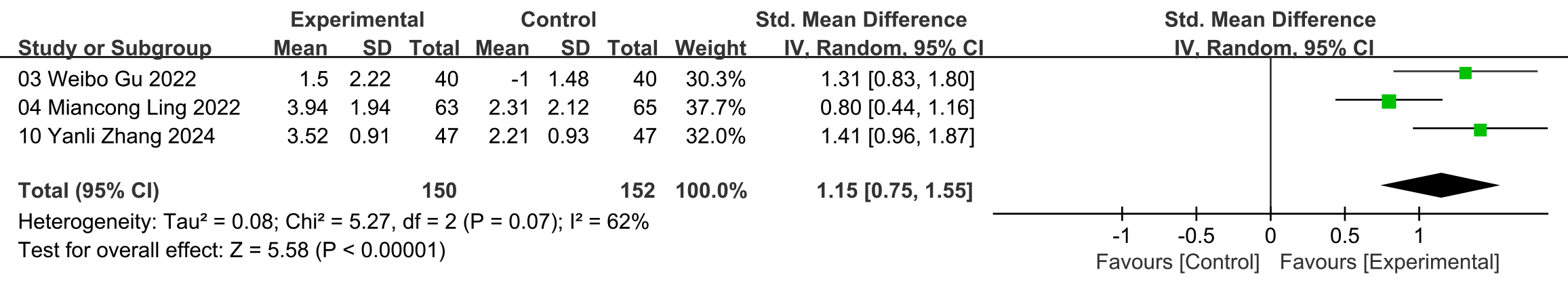

Supplement: Supplementary file 1 [file Supplementary_file_1.zip › Supporting Information/3.Supplementary figure/Figure S4. Forest plot of short physical performance battery (SPPB) with random-effects model.tif]

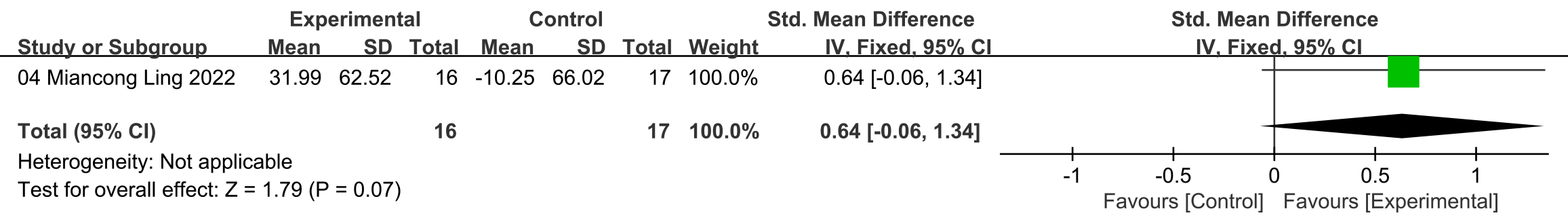

Supplement: Supplementary file 1 [file Supplementary_file_1.zip › Supporting Information/3.Supplementary figure/Figure S5. Forest plot of the 6-minute walk test with random-effects model.tif]

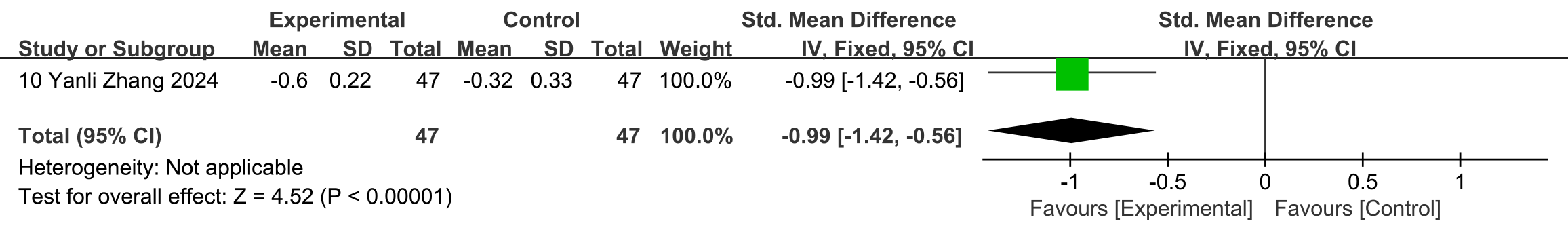

Supplement: Supplementary file 1 [file Supplementary_file_1.zip › Supporting Information/3.Supplementary figure/Figure S6. Forest plot of C-reactive proteins with random-effects model.tif]

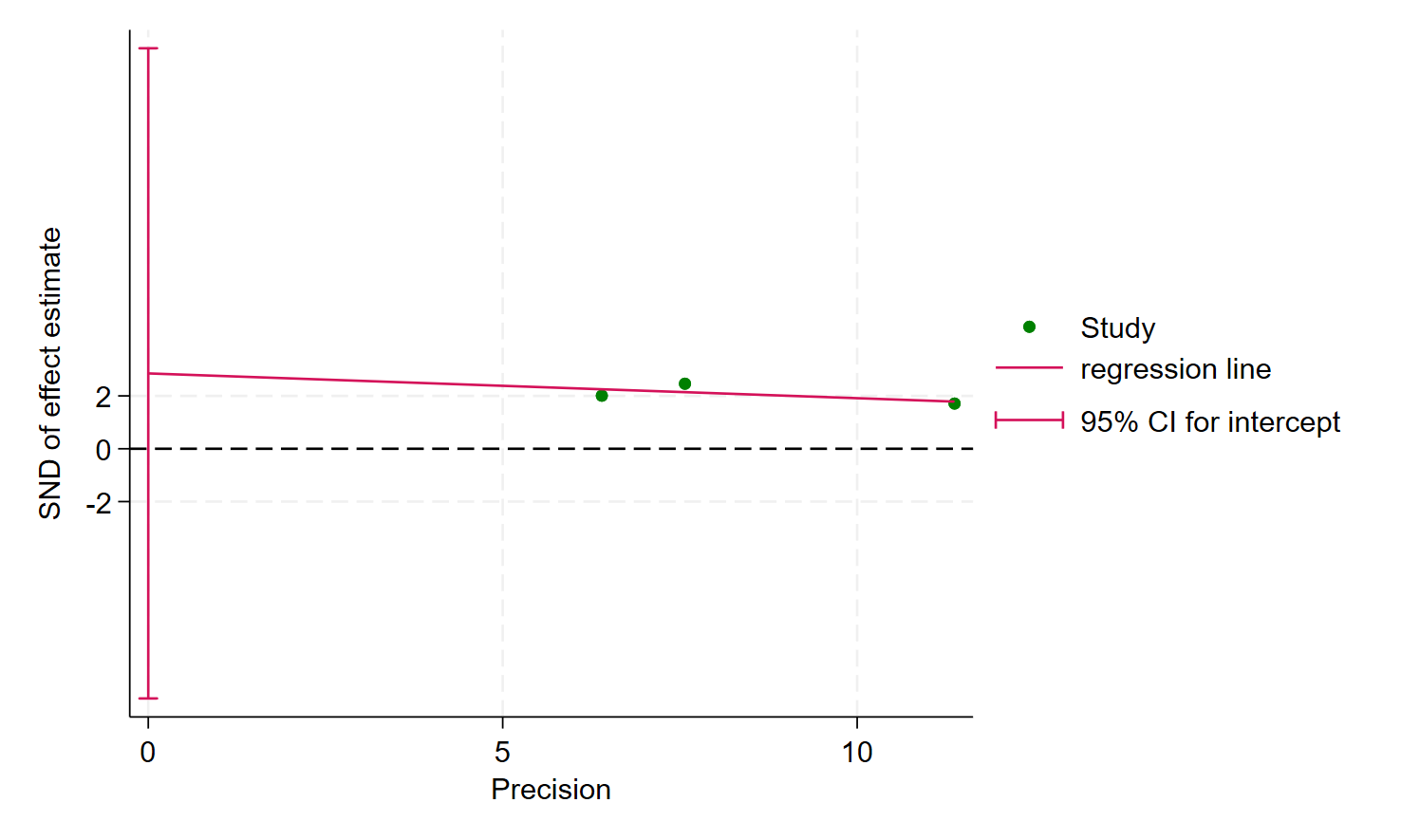

Supplement: Supplementary file 1 [file Supplementary_file_1.zip › Supporting Information/3.Supplementary figure/Figure S7. Egger’s test plot of total efficiency.tif]

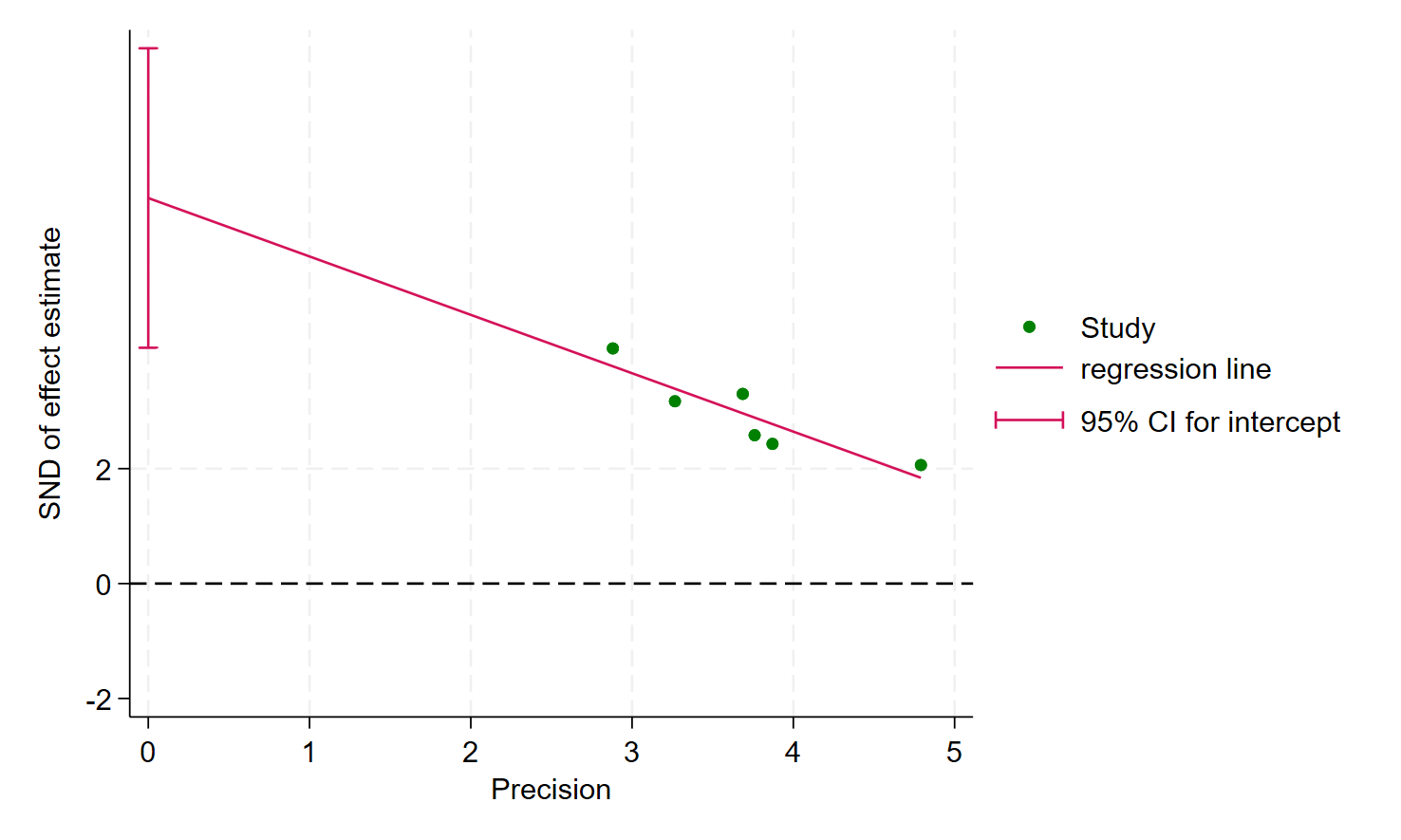

Supplement: Supplementary file 1 [file Supplementary_file_1.zip › Supporting Information/3.Supplementary figure/Figure S8. Egger’s test plot of muscle mass.tif]

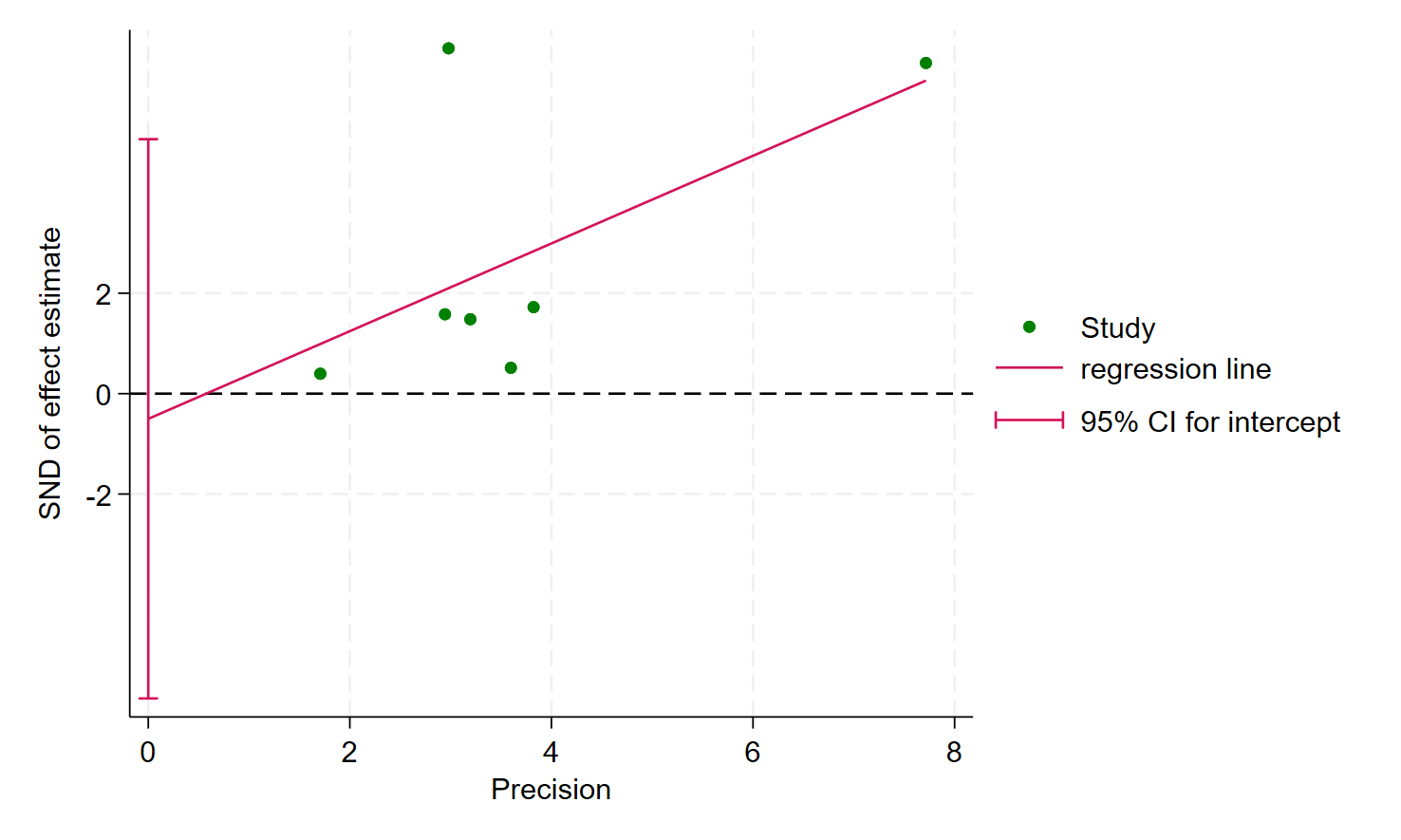

Supplement: Supplementary file 1 [file Supplementary_file_1.zip › Supporting Information/3.Supplementary figure/Figure S9. Egger’s test plot of grip strength.tif]
